# Maternal inhalation of carbon black nanoparticles induces neurodevelopmental changes in mouse offspring

**DOI:** 10.1186/s12989-018-0272-2

**Published:** 2018-09-10

**Authors:** Masakazu Umezawa, Atsuto Onoda, Irina Korshunova, Alexander C. Ø. Jensen, Ismo K. Koponen, Keld A. Jensen, Konstantin Khodosevich, Ulla Vogel, Karin S. Hougaard

**Affiliations:** 1 National Research Centre for the Working Environment, Lersø Parkallé 105, DK-2100 Copenhagen Ø, Denmark; 2 Department of Micro- and Nanotechnology, Technical University of Denmark, Lyngby, Denmark; 3 Institute of Public Health, University of Copenhagen, Copenhagen K, Denmark; 4 Research Institute for Science and Technology, Organization for Research Advancement, Tokyo University of Science, Noda, Chiba, Japan; 5 Department of Materials Science and Technology, Faculty of Industrial Science and Technology, Tokyo University of Science, Katsushika, Tokyo, Japan; 6 Department of Hygienic Chemistry, Graduate School of Pharmaceutical Sciences, Tokyo University of Science, Noda, Chiba Japan; 7 Japan Society for the Promotion of Science, Chiyoda, Tokyo, 102-0083 Japan; 8 Biotech Research and Innovation Centre (BRIC), Faculty of Health, University of Copenhagen, Copenhagen K, Denmark

**Keywords:** Carbon black, Maternal exposure, Prenatal exposure, Pregnancy, Brain, Nanoparticle, Behaviour, Astrocyte, Neurodevelopment, Developmental neurotoxicity

## Abstract

**Background:**

Engineered nanoparticles are smaller than 100 nm and designed to improve or creating even new physico-chemical properties. Consequently, toxicological properties of materials may change as size reaches the nm size-range. We examined outcomes related to the central nervous system in the offspring following maternal inhalation exposure to nanosized carbon black particles (Printex 90).

**Methods:**

Time-mated mice (NMRI) were exposed by inhalation, for 45 min/day to 0, 4.6 or 37 mg/m^3^ aerosolized carbon black on gestation days 4–18, i.e. for a total of 15 days. Outcomes included maternal lung inflammation (differential cell count in bronchoalveolar lavage fluid and *Saa3* mRNA expression in lung tissue), offspring neurohistopathology and behaviour in the open field test.

**Results:**

Carbon black exposure did not cause lung inflammation in the exposed females, measured 11 or 28–29 days post-exposure. Glial fibrillary acidic protein (GFAP) expression levels were dose-dependently increased in astrocytes around blood vessels in the cerebral cortex and hippocampus in six weeks old offspring, indicative of reactive astrogliosis. Also enlarged lysosomal granules were observed in brain perivascular macrophages (PVMs) in the prenatally exposed offspring. The number of parvalbumin-positive interneurons and the expression levels of parvalbumin were decreased in the motor and prefrontal cortices at weaning and 120 days of age in the prenatally exposed offspring. In the open field test, behaviour was dose-dependently altered following maternal exposure to Printex 90, at 90 days of age. Prenatally exposed female offspring moved a longer total distance, and especially males spent significantly longer time in the central zone of the maze. In the offspring, the described effects were long-lasting as they were present at all time points investigated.

**Conclusion:**

The present study reports for the first time that maternal inhalation exposure to Printex 90 carbon black induced dose-dependent denaturation of PVM and reactive astrocytes, similarly to the findings observed following maternal exposure to Printex 90 by airway instillation. Of note, some of the observed effects have striking similarities with those observed in mouse models of neurodevelopmental disorders.

**Electronic supplementary material:**

The online version of this article (10.1186/s12989-018-0272-2) contains supplementary material, which is available to authorized users.

## Background

Nanotechnology provides benefits in the development of new products and uses, but also evokes a potential for new health risks through occupational, consumer and environmental exposure to nanomaterials [1]. It follows that the toxicity of nanomaterials needs investigation to enable introduction of adequate levels of protection, and that developmental toxicity should be an integrated part of the toxicological dossier [2–4]. Toxicity studies of engineered nanomaterials, such as carbon black nanoparticles (CB-NP), may furthermore provide insight into the potential health impacts of carbonaceous soot particles in ambient air pollution, especially those in the ultrafine and fine range [5].

When highly dispersed NP are inhaled, most will deposit deep in the airways and clear away only slowly [6]. A small fraction may traverse biological barriers, including the air-blood interface in the lungs [7, 8] and the blood-placental barrier [9, 10]. Recently, Printex 90 CB and TiO_2_ NP administered via the airways were shown to translocate to the liver [11], demonstrating their translocation across the lung-blood barrier. NP therefore have the potential to accumulate in the offspring, as first demonstrated in vivo following subcutaneous administration of TiO_2_ NP to pregnant mice and their subsequent detection in offspring brain and testes up to six weeks after birth [12]. Although the potential for translocation across the placenta may vary between particle types [13], it is possible that NP may affect foetal development adversely due to direct interaction of particles with foetal tissues. The underlying mechanism could be local generation of reactive oxygen species, a generally accepted causative factor in the toxicity of nanomaterials [14–16]. In the brain, direct interaction of particles with specific subpopulations of cells (microglia and astrocytes) may initiate production of free radicals, and hence promote oxidative stress, and induce inflammation, to which the brain is most sensitive during development [17]. Effects of inhaled particles have also been proposed to be mediated indirectly, especially via the resulting maternal lung inflammation and subsequent circulation of cytokines or other secondary inflammatory messengers [3, 18]. It has been previously shown that maternal inflammation may prime early alterations in the inflammatory response systems, which in turn can disrupt development and maturation of the central nervous system [19]. Activation of the maternal immune response during pregnancy, by injection of influenza virus, polyinosinic-polycytidilic acid (poly(I:C)), or lipopolysaccharide (LPS), are established mouse models that demonstrate the causal relationship between maternal inflammation and changes in brain development and cognitive function in the offspring [20–22]. Both mouse models and epidemiological studies demonstrate correlations between maternal inflammation and risk of developing schizophrenia or autism spectrum disorder in the offspring [23–25]. Among the different types of neurons, particularly the parvalbumin-positive (PV+) interneurons in the cortex and hippocampus of the affected offspring seem to be vulnerable to oxidative stress caused by maternal inflammation [26–29]. This has furnished the theory that oxidative stress could be a common pathological mechanism in impairment of PV+ interneurons in among others schizophrenia [26]. Hence, the developing nervous system may be especially sensitive to maternal particle exposure [17, 30], via direct or indirect mechanistic pathways.

Carbon black is a high-volume material that is widely used as a black pigment. The chemically pure carbon black particle Printex 90 has often been applied as a model particle for the primary traffic-generated carbonaceous soot particles in toxicological studies of particulate air pollution [31–34].

In developmental studies, CB-NP airway exposure of pregnant mice has been shown to increase reactivity of the offspring immune system [35] and to induce brain perivascular abnormalities (astrogliosis) in the offspring brain in a dose-dependent manner following intranasal instillation [36, 37]. Specifically, perivascular macrophages (PVM) presented with enlarged granules, and increased levels of glial fibrillary acidic protein (GFAP) were observed in astrocytes attached to these enlarged granules [36]. Increased GFAP expression period has also been shown in the brain (amygdala) of mice exposed to concentrated ambient particles during the neonatal period [38]. The perivascular pathology and astrogliosis were furthermore accompanied by accumulation of β-sheet-rich waste proteins [37]. Interestingly, GFAP is often up-regulated in astrocytes under conditions of inflammation [39, 40]. The potential behavioural consequence of the observed neuropathology has not been assessed, but a previous study of Printex 90 reported a changed profile in offspring open field behaviour following maternal instillation of carbon black during gestation [41].

Of note, in the studies reporting perivascular abnormalities the pregnant mice were exposed by intranasal instillation [36, 37]. This mode of exposure is often applied in proof of principle studies, as it provides high control over the dose level. The particles are however delivered as a bolus, i.e. at a much higher dose rate than under realistic inhalation conditions. Inhalation studies are therefore required to establish the effects of particle exposure under conditions with a higher resemblance to real life, to reduce data gaps in risk assessment.

Based on the above, we hypothesized that maternal inhalation exposure to Printex 90 CB, in the offspring would induce dose-dependent brain perivascular abnormalities, characterized by astrogliosis and enlargement of granules in perivascular macrophages, as previously observed following exposure by intranasal instillation; change behaviour in the open field test; and that development and maturation of interneurons would be affected.

## Methods

### Animals

Sixty time-mated, nulliparous female mice (NMRI, Taconic Europe, Ejby, Denmark) arrived at gestation day (GD) 3 and were randomly distributed to clear polypropylene cages (open cage system, 1296D Eurostandard Type 3) holding five mice each and housed in an open cage system under controlled environmental conditions (light from 6 a.m. to 6 p.m., temperature 21 °C ± 2 °C and humidity 50% ± 5%) with aspen bedding (Tapvei, Estonia), enrichment consisting of mouse house (80-ACRE011, Techniplast, Italy), small aspen blocks (Tapvei, Estonia) and nest material (Enviro Dri, Lillico, Biotechnology, UK), and ad libitum access to feed (Altromin 1314) and tap water. Upon arrival, the animals were allocated randomly to three groups of each 20 time-mated females. Procedures complied with EC Directive 86/609/EEC and Danish regulations on experiments with animals (Permit 2015–15–0201-00569), and the research protocol was approved by the local Animal Welfare Committee prior to study.

### Material characterization

This study used carbon black Printex 90 nanoparticles (a gift from Degussa-Hüls, Frankfurt, Germany). Printex 90 has been characterized previously, as summarized in [42]. They have an average primary particle size of 14 nm, a specific surface area of 182–338 m^2^/g [43], and very low levels (< 1%) of organic and inorganic impurities. Printex 90 has the intrinsic potential to generate reactive oxygen species, is inflammogenic and mutagenic. Carbon black is furthermore classified by the International Agency for Research on Cancer (IARC) as possibly carcinogenic to humans. Results from Limulus Amebocyte Lysate Testing of our batch of Printex 90 have previously shown that the endotoxin level was below the upper tolerance limit for human and veterinary drugs (5 EU/kg body weight/hour or 330 pg/kg body weight /hour) [42].

### Exposure

The time-mated mice were exposed whole body, as previously described [44, 45], from GD 4 to 18 for 45 min/day, to either HEPA-filtered clean air (0 mg Printex 90/m^3^) or target concentrations of 4.6 or 37 mg/m^3^ of Printex 90, in the following referred as control, low and high exposure, respectively. All animals were exposed on all days (15 consecutive days in total). For the low exposure, the source was active for 15 min followed by 7.5 min of inactivity, and the cycle was then repeated for the total exposure time of 45 min. After exposure, females were observed for signs of toxicity and returned to their cages less than 5 min after the end of exposure. The estimated daily deposited doses achieved in mice during 45 min of exposure to 4.6 and 37 mg/m^3^ correspond to approximately 1 and 8 h of exposure at the 8 h time-weighted occupational exposure limit of 3.5 mg/m^3^ in Denmark, respectively [46]. During exposure, the animals were placed in separate rooms of a cylindrical wire mesh cage (∅ 29 cm, height 9 cm) with twelve rooms arranged in radial partitions. The wire-mesh cage was placed in an 18 L spheroidal chamber with a stainless steel rim and upper and lower spheres in fully transparent glass and designed to provide an evenly distributed exposure atmosphere [See Additional file 1]. Printex 90 was aerosolized at a pressure of 5 bar using a venturi-type rotating disc microfeeder (Fraunhofer Institute für Toxicologie und Aerosolforschung, Hannover, Germany) and directed through an aerosol mixing and sedimentation glass-tube to the exposure chamber at a total volume-flow of 20 L/min.

### Exposure monitoring

Mass-concentrations of total suspended dust were followed gravimetrically by filter sampling to adjust and maintain the concentrations of 4.6 or 37 mg/m^3^ in the chamber. Exposure air was continuously sampled on Millipore Fluoropore filters (∅ 2.5 cm; pore size 0.45 μm) at an airflow of 2 L/min, using Millipore cassettes. Acclimatized filters were pre-weighed on a Sartorius Microscale (Type M3P 000 V001). Final gravimetric data were obtained on filters acclimatized (50%RH and 20°C) for at least for at least 24 h (LOD 0.007 mg/filter).

Particle number and aerodynamic particle size distributions in the exposure atmosphere were measured in 14 size channels between 6 nm and 10 μm with 1 s intervals using an Electrical Low Pressure Impactor (ELPI+, Dekati Ltd., Tampere, Finland). Controls were monitored using a condensation particle counter TSI model 3007 (CPC; TSI Inc., Shoreview, MN, USA) with a detection range from ca. 10 nm to > 1 μm.

### Parturition and lactation

After the last exposure, on GD 18, females were singly housed. Delivery was expected on GD 20, and designated postnatal day (PND) 0. The dam stayed with the pups until weaning at PND 21. Dams without litters were euthanized at PND 9 and dams with litters at PND 26–27. Animals were anaesthetized by intraperitoneal injection of a cocktail of Zoletil Forte 250 mg/ml, Rompun 20 mg/ml, Fentanyl 50 μg/ml in sterile isotone saline, at 0.1 ml/10 g body weight, and sacrificed by withdrawal of heart blood. All dams were subjected to bronchoalveolar lavage (BAL) following anaesthesia.

### Allocation of offspring

A maximum of one male and one female offspring per litter was assessed for any one outcome to avoid litter effects. For testing in the open field test, one male and one female per litter were randomly chosen for behavioural testing at weaning (Table 1) and housed as described. For GFAP immunohistochemistry, one male per litter was sacrificed by decapitation at six weeks of age, as this time point was used in the prior instillation studies of perivascular macrophages [36, 37]. The brain was dissected, fixed in 4% formaldehyde, transferred to 30% sucrose with 1% sodium azide 24 h later and stored at 4 °C until use. The brains for analysis of PV+ interneurons were collected from male and female offspring at weaning on PND 25. In addition, brains were collected from adult female offspring that had undergone open field testing and given birth to a 2nd generation, to enable study of the persistence of effects. Females were anaesthetized and sacrificed as described above, and the dissected brains were fixed in 4% formaldehyde until use. All assessments were performed by experimenters blinded to the exposure status of the animals.

**Table 1:** Overall design and group sizes for the different outcomes

Group	Control	Low	High
Mass concentration (mg/m^3^)	Below detection limit	4.79 ± 1.86	33.87 ± 14.77
Particle number conc. (cm^−3^)	4 ± 4	3.59 ± 2.48∙10^5^	2.12 ± 0.69∙10^6^
Exposure period, all females	4–18	4–18	4–18
Exposed time-mated females	20	20	20
Dams delivering litters^a^	14	16	15
Litter size	10.9 ± 3.2	11.3 ± 3.3	11.1 ± 2.7
Differential cell counts			
- Non-littering females (PND 9)	4	4	5
- Littering females (PND 26–27)^b^	5	9	13
Saa3 in lung tissue			
- Non-delivering females (PND 9)	6	4	5
- Delivering females (PND 26–27)	11	12	13
Offspring outcomes^+^			
- GFAP in brain tissue (6 weeks)	6 males	5 males	4 males
- PV+ interneurons (PND 25)^c^	6 females and 5 males	–	7 females and 3 males
- PV+ interneurons (PND 120)^d^	6 females	–	6 females
- Open field females/males^d^	9/11	11/10	13/13

^a^Females that did not deliver a litter was killed at PND 9 and inflammatory outcomes assessed. ^b^For differential cell counts, some slides were accidentally processed with too few cells and could not be included in the analysis, resulting in small group sizes. ^a^A maximum of one male and one female was used in any one test. ^c^The density of PV+ interneurons and expression of PV were similar for males and females within each condition and were combined prior to statistical analysis. ^d^The females assessed for PV+ interneurons had been tested in the open field at three months of age

### BAL preparation and analyses

We used BAL fluid (BALF) total cell and differential counts to indicate lung inflammation, 11 (time-mated females without litters) and 28–29 days post-exposure (dams with litters). BAL was performed by flushing the lungs twice with 0.8 ml sterile 0.9% sodium chloride (NaCl) through the trachea to obtain BALF. BALF was stored on ice until centrifugation at 400×*g* for 10 min at 4 °C. BAL cells were resuspended in 100 μl medium (HAM F-12 with 1% penicillin/streptomycin and 10% foetal bovine serum). The total number of living and dead cells in BAL was determined by NucleoCounter NC-200TM (Chemometec, Denmark) from 20 μl diluted cell suspension following the manufacturer’s protocol. Cell composition was determined from 40 μl of cell resuspension, following centrifugation at 55×*g* for 4 min in Cytofuge 2 (StatSpin, TRIOLAB, Brøndby, Denmark) on to a microscope glass slide to be fixated for 5 min in 96% ethanol. All slides were stained with May-Grünwald-Giemsa, randomized and blinded, before counting 200 cells/sample under light microscope [47].

### *Saa3* mRNA expression analysis

RNA was isolated from lung tissue (16–20 mg) on Maxwell® 16 (Promega, USA) using Maxwell® 16 LEV simply RNA Tissue Kit (AS1280, Promega, USA) according to the manufacturer’s protocol. RNA was eluted in 50 μl nuclease free (DEPC) water. cDNA was prepared from DNase treated RNA using Taq-Man® reverse transcription reagents (Applied Biosystems, USA) as described by manufacturer’s protocol. Total RNA and cDNA concentrations were measured on NanoDrop 2000c (ThermoFisher, USA). The *Saa3* mRNA levels were determined using real-time RT-PCR with 18S RNA as reference gene as previously described [48]. In brief, each sample was run in triplicates on the ViiA7 Real-Time PCR (Applied Biosystems, USA). *Saa3* primers and probe sequences were: *Saa3*forward: 5’ GCC TGG GCT GCT AAA GTC AT 3′, *Saa3*reverse: 5’ TGC TCC ATG TCC CGT GAA C 3′ and *Saa3* probe: 5’ FAM-TCT GAA CAG CCT CTC TGG CAT CGC T-TAMRA 3′. In all assays, TaqMan predeveloped mastermix (Applied Biosystems, USA) was used. *Saa3* and 18S RNA levels were quantified in triplicates in separate wells. The relative Saa3 expression levels were calculated by the comparative method 2^-ΔCt^. mRNA measurements were excluded if the 18S RNA content fell outside the linear range in which the PCR was found to be quantitative defined by the validation experiments [34]. Negative controls, where RNA had not been converted to cDNA (no template control), were included in each run. One sample, the plate control, was included in all Real-Time PCR analyses. The day-to-day variation was 2% for the plate control.

### Double-staining for GFAP and periodic acid Schiff (PAS) positive granules

Brains from 6 week old male offspring (*n* = 4–6) were used for double-staining of GFAP and PAS-positive granules (PAS-GFAP immunohistochemistry). PAS-GFAP immunohistochemistry allows assessment of the morphology of blood vessels including granules in the brain perivascular macrophages (PVMs) and astrocytes around blood vessels as well as their relationship because both are visualized in the same section. The fixed right brain hemisphere was embedded in Tissue-Tek OCT compound (Sakura Finetek Japan Co., Ltd., Tokyo, Japan), frozen, and then cut into 10-μm sections. Cardiac perfusion was not performed to avoid vascular breakdown and denaturation of the blood vessels by pressure of the perfusion. Visualization of GFAP and PAS-positive granules was performed on the sections using the appropriate antibodies and an avidin–biotin-peroxidase method. After blocking endogenous peroxidase by preincubation with 10% normal horse serum, sections were incubated in primary rabbit polyclonal anti-GFAP antibody (DakoCytomation) diluted 1:1000 in PBS containing 0.1% Trion X (PBS-Tx) for 16 h at 4 °C. After rinsing 3 times for 5 min per rinse with PBS-Tx, sections were further incubated in secondary biotinylated donkey anti-rabbit IgG (Chemicon, Temecula, CA, USA; 1:1000) for 120 min at room temperature and rinsed 3 times for 5 min per rinse with PBS-Tx. Sections were then treated with 1% periodic acid solution for 3 min, rinsed with distilled water for 1 min, and soaked in cold Schiff reagent for 60 min. Next, sections were soaked in sulphurous acid solution 3 times for 3 min per soak and then rinsed with distilled water for 1 min. Finally, sections were treated with an avidin-biotin-peroxidase complex (Vectastain ABC peroxidase kit, Vector Laboratories Inc., CA, USA; 1:400) for 120 min and reacted in a solution of 0.02% 3,3′-diaminobenzidene (DAB) in 0.1 M Tris–HCl buffer (pH 7.6) and 0.01% H_2_O_2_ for 20 min to detect peroxidase activity. GFAP immunoreactivity localized to the astrocytic cytoplasm was visible as light-brown staining. Sections were then washed in PBS, dehydrated in graded alcohol, cleared in xylene, and coverslips were applied with permount mounting medium (Thermo Fisher Scientific Inc., MA, USA). Fifty sections (total 300 μm) from the longitudinal fissure of the cerebrum along the sagittal plane were prepared from each mouse. One in every 5 sections (total 10 sections per mouse) was subjected to PAS-GFAP immunohistochemistry and analysis. These sections were observed by a BX41 microscope (Olympus, Co., Tokyo, Japan) equipped with a digital camera (Olympus). All slides were coded before scoring. The slides were scored by two different investigators who were blinded in regard to the code of the slides. Each investigator ranked the slides according to the intensity of staining; low rank corresponded to the least intense staining. The results are expressed as rank, based on the mean of the ranks obtained by the two different investigators.

### Quantitation of GFAP in immunofluorescently stained brain sections

Immunofluorescence is superior to quantify the expression level of a target protein, therefore the 10-μm brain sections were also subjected to immunofluorescence for quantitative evaluation of GFAP protein expression. Sections were blocked with 10% normal horse serum for 1 h at room temperature and then incubated with primary goat polyclonal anti-GFAP antibody (Abcam, Cambridge, UK; 1:500) diluted 1:1000 in PBS for 16 h at 4 °C. After rinsing 3 times for 5 min per rinse with PBS, sections were further incubated with secondary Dylight 488-conjugated donkey anti-goat IgG (Rockland Immunochemicals Inc., PA, USA; 1:1000) for 120 min at room temperature, and rinsed 3 times for 5 min per rinse with PBS and twice for 5 min per rinse with distilled water, and nuclei were counter-stained with Hoechst 33342 (Dojindo Laboratories, Kumamoto, Japan). Digital images were acquired by fluorescence microscopy (Biorevo BZ-9000, Keyence Co., Osaka, Japan) from three randomly (with > 30 μm interval) chosen hippocampal and cerebral sections from each brain and stored as TIFF files. Forty-five selected images (control: 12, low-dose: 18, high-dose: 15) were used for quantification of the GFAP expression level. The files were then opened in ImageJ software (National Institute of Health, MD, USA) and further processed to quantitatively assess GFAP expression level. To quantify the pixels stained with Dylight 488, green channel images were converted to grey scale (Image J command, Image: Colour: Split Channels). The grayscale levels were then expanded linearly so that the values 40–80 (cerebral cortex) and 30–150 (hippocampus) fell into 0–255 (ImageJ command, Image: Adjust: Brightness/Contrast). The grayscale threshold was set (ImageJ command, Image: Adjust: Threshold) so that all pixels above the chosen grayscale value (70) were included for measurement and those below excluded. A common threshold level was set for each image to prevent intra-assay variations. Then the fraction of white pixels, representing GFAP expression, was quantified by histogram analysis (ImageJ command, Analyse: Measure after setting “Area” and “Limited to Threshold” in Image J command, Analyse: Set Measurement). During all preparations and analyses, the experimenter was blinded to the exposure status of the animals. The mean value of GFAP immunofluorescence from the three brain sections/mouse was calculated and used in the statistical analysis.

### Analysis of distribution of PV+ interneurons

We aimed to characterize maturation of PV+ interneurons in the prefrontal and motor cortices, some of the main regions affected in neurodevelopmental disorders [19, 49–52]. Immunohistochemistry of inhibitory interneurons in brains fixed in 4% formaldehyde was performed as described previously [53, 54]. Brains from control and high-dose prenatally exposed animals were analysed at PND 25 (n_females_ = 6–7; n_males_ = 3–5) and PND 120 (n_females_ = 6). Coronal brain sections (50 μm) were cut with a vibratome (Leica VT1000S, Leica, Germany) and immunohistochemistry was carried out on free-floating sections. Sections were blocked in 0.4% Triton and 5% BSA in PBS for 30 min at room temperature and incubated overnight with rabbit anti-EGFP antibody (Invitrogen, Germany; 1:1000, 0.2% Triton, 5% BSA in PBS) at 4°C followed by goat anti-rabbit 488 secondary antibody (Invitrogen, Germany; 1:10000, 0.2% Triton, 5% BSA in PBS) at room temperature. As the last step of immunohistochemistry, 4′,6-diamidino-2-phenylindole (DAPI) was used for nuclear staining (1:1000 in PBS, 10 min at room temperature). Sections were mounted onto slides with Immumount (ThermoScientific, Germany). Images for quantification of PV+ interneurons in the prefrontal and motor cortices were acquired on a confocal microscope Leica SP8 (Leica Microsystems, Germany). Single plane images were made using a 20× objective. Microscope parameters for image acquisition were the same for all the images. Image analysis was done with Fiji/ImageJ software [55].

### Behaviour in the open field test

This test was included as previous studies on carbon black as well as of other nanomaterials have observed altered behaviour in the open field [41, 45]. Investigations were performed during the light period, as described [45]. Animals were transferred to the experimental room ½ an hour before testing of the first animal. Prenatally exposed and control animals were tested alternately. The observer was blinded to the prenatal exposure status of the animals, and the same observer tested all animals. Locomotive activity was assessed for 3 min at 90 days of age in a circular open field (∅ 100 cm) of black plastic with tall sides. This age was chosen to ease comparison with a previous study of gestational particle exposure [45]. Trials commenced in the centre of the field and the location of the animal was registered by Noldus Ethovision XT version 5. The tracking device calculated total ambulation, which was subsequently split into three time-bins of 1 min to test for habituation. Duration in the central and the outer peripheral zone of the field, as well as the number of crossings from the outer to the central zone were extracted.

### Statistics

*P*-values < 0.05 were considered statistically significant and a maximum of one male and one female per litter were used in any one test. BAL cell and differential counts and *Saa3* mRNA levels were analysed by two-way ANOVA, with Exposure level and Day after exposure as factors, and open field data by two-way ANOVA, with Exposure level and Sex as factors (Hougaard et al. 2005), followed by Fisher’s Least-Statistical-Difference test when appropriate (SYSTAT Software Package 9). GFAP immunofluorescence was analysed by one-way ANOVA followed by Tukey-Kramer multiple comparisons test (Excel Statistics 2012, Social Survey Research Information, Tokyo, Japan). For PV+ interneurons, the datasets with more than 2 groups were compared using one-way ANOVA followed by Bonferroni multiple comparison test; the data sets with 2 groups were compared by Student’s t-test. Since the density of PV+ interneurons and expression of PV were similar for males and females at PND25 within each condition, we combined the data from both sexes and then statistically analysed them according to the description above (GraphPad Prism version 6.00 for Mac OS X (GraphPad Software, USA, www.graphpad.com).

## Results

### Exposure characteristics

**Fig. 1:**
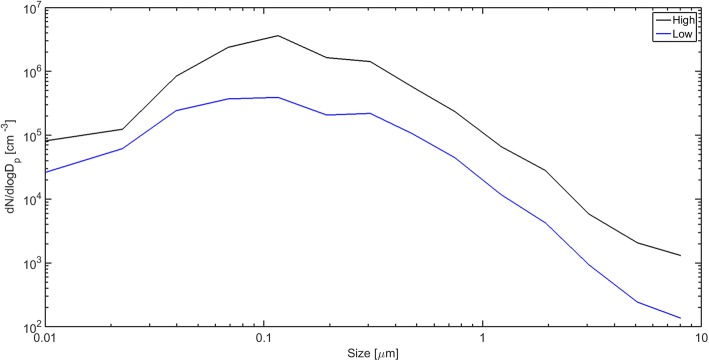
Average particle size distributions measured by the ELPI+ for the low and high exposure groups (sham exposure level for control animals not shown)

Filter measurements demonstrated that the time-mated animals were exposed to a mean total suspended particle mass concentration: below the detection limit (LOD 0.08 mg/m^3^), 4.79 ± 1.86, or 33.87 ± 14.77 mg/m^3^ carbon black for the control, low, and high exposure level, respectively. The corresponding median particle number concentrations in the exposure atmosphere with semi interquartile deviation were 4 ± 4 cm^− 3^, 3.59 ± 2.48∙10^5^ cm^− 3^, and 2.12 ± 0.69∙10^6^ cm^− 3^ carbon black for the control, low, and high exposure level, respectively [See Additional file 2]. The large variation for the low exposure level primarily owes to the exposure regime, with the source being active for only two thirds of the exposure period. The measured aerosol number size distribution was found to be bi-modal, peaking at 100 and 300 nm, see Fig. 1.

### Lung inflammation in time-mated females

**Fig. 2:**
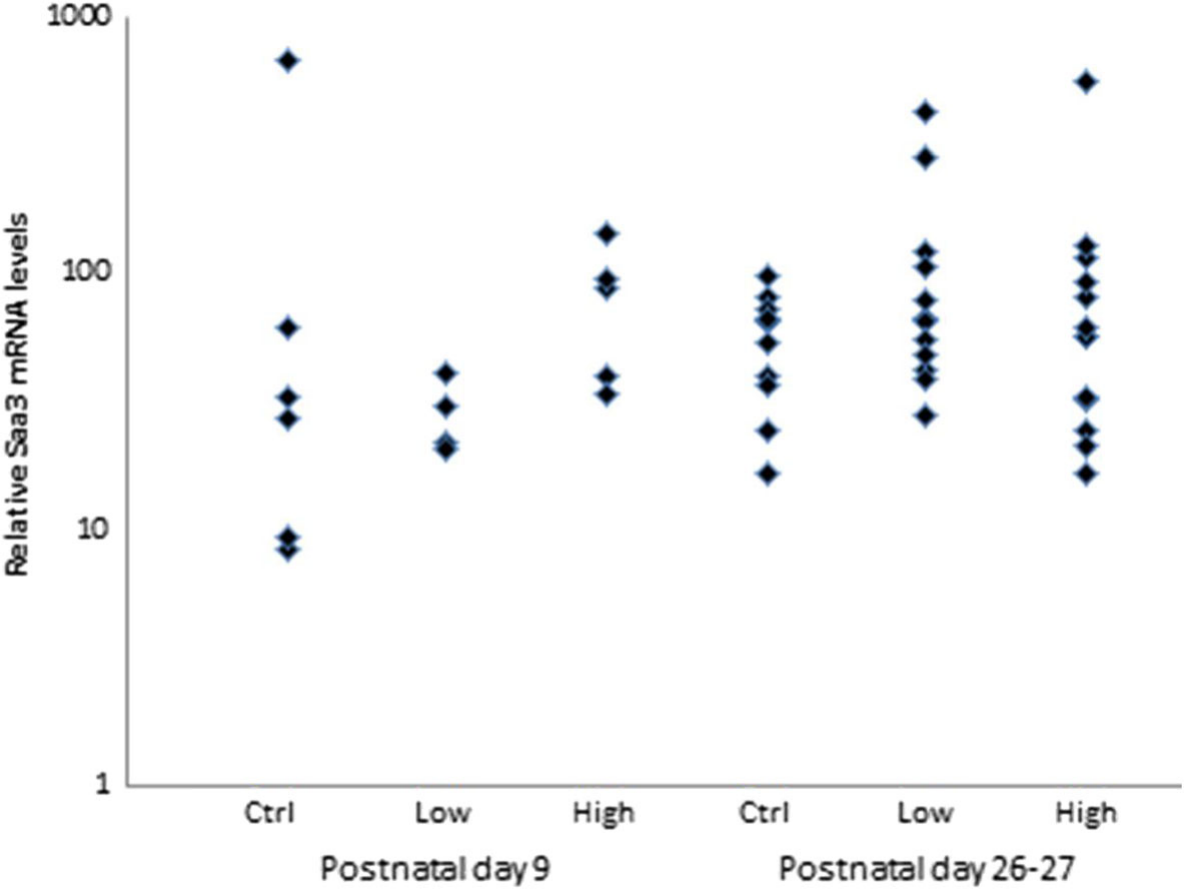
Relative *Saa3* mRNA levels in lungs from time-mated mice exposed to Printex 90 by inhalation, at PND 9 and PND 26–27 (corresponding to 11 and 28–29 days after termination of exposure). Expression of mRNA was normalized to 18S rRNA

Lung inflammation in exposed time-mated females was evaluated by total and differential cell counts in BALF and by analysis of mRNA expression of Serum Amyloid A (Saa3) in lung tissue at PND 9 in dams without litters and in dams with a litter at PND 26–27, corresponding to 11 and 28–29 days after the last exposure, respectively. No difference was observed between groups for total cell counts. For the differential cell counts, some slides were accidentally processed with less than 200 cells and could not be included in the analysis, resulting in *n* = 4–13 (Table 1). The overall analysis revealed no statistically significant differences between groups for any cell population (Table 2). *Saa3* mRNA levels in Printex 90 exposed females were also not statistically significantly different from air exposed controls, although a non-significant dose-effect relationship was observed at PND 9 (Fig. 2).

**Table 2:** BAL fluid cell composition, 11 and 28–29 days after termination of inhalation exposure to Printex 90 for 15 days

		Absolute cell numbers		
	Exposure level of Printex 90	Control	3.59 mg/m^3^	33.87 mg/m^3^
11 days	*N*	4	4	5
	Neutrophils (×10^5^)	0.4 ± 0.4	0.4 ± 0.5	1.4 ± 2.6
	Macrophages (×10^5^)	8.0 ± 2.7	7.6 ± 8.9	7.0 ± 4.2
	Lymphocytes (× 10^3^)	3.1 ± 4.2	3.1 ± 3.9	0.8 ± 1.7
	Total BAL cells (×10^5^)	9.2 ± 2.8	8.6 ± 0.5	11.3 ± 3.5
	Epithelial cells (×10^5^)	0.7 ± 0.4	0.6 ± 0.3	0.7 ± 0.5
	Dead cells (×10^5^)	0.9 ± 0.5	1.1 ± 0.8	1.2 ± 0.4
28–29 days	*N*	5	9	13
	Neutrophils (×10^5^)	1.5 ± 1.8	1.0 ± 0.9	1.2 ± 1.0
	Macrophages (×10^5^)	13.0 ± 2.7	11.9 ± 4.1	9.8 ± 1.6
	Lymphocytes (×10^3^)	15.2 ± 16	12.5 ± 6.5	6.4 ± 6.2
	Total BAL cells (×10^5^)	15.4 ± 4.6	13.8 ± 4.1	11.7 ± 2.0
	Epithelial cells (×10^5^)	0.8 ± 0.4	0.8 ± 0.3	0.6 ± 0.3
	Dead cells (×10^5^)	1.3 ± 1.1	1.4 ± 0.7	1.1 ± 0.8

Data presented as mean number ± SD

### Histopathological analysis of PVMs and astrocytes in 6 weeks old male offspring

**Fig. 3:**
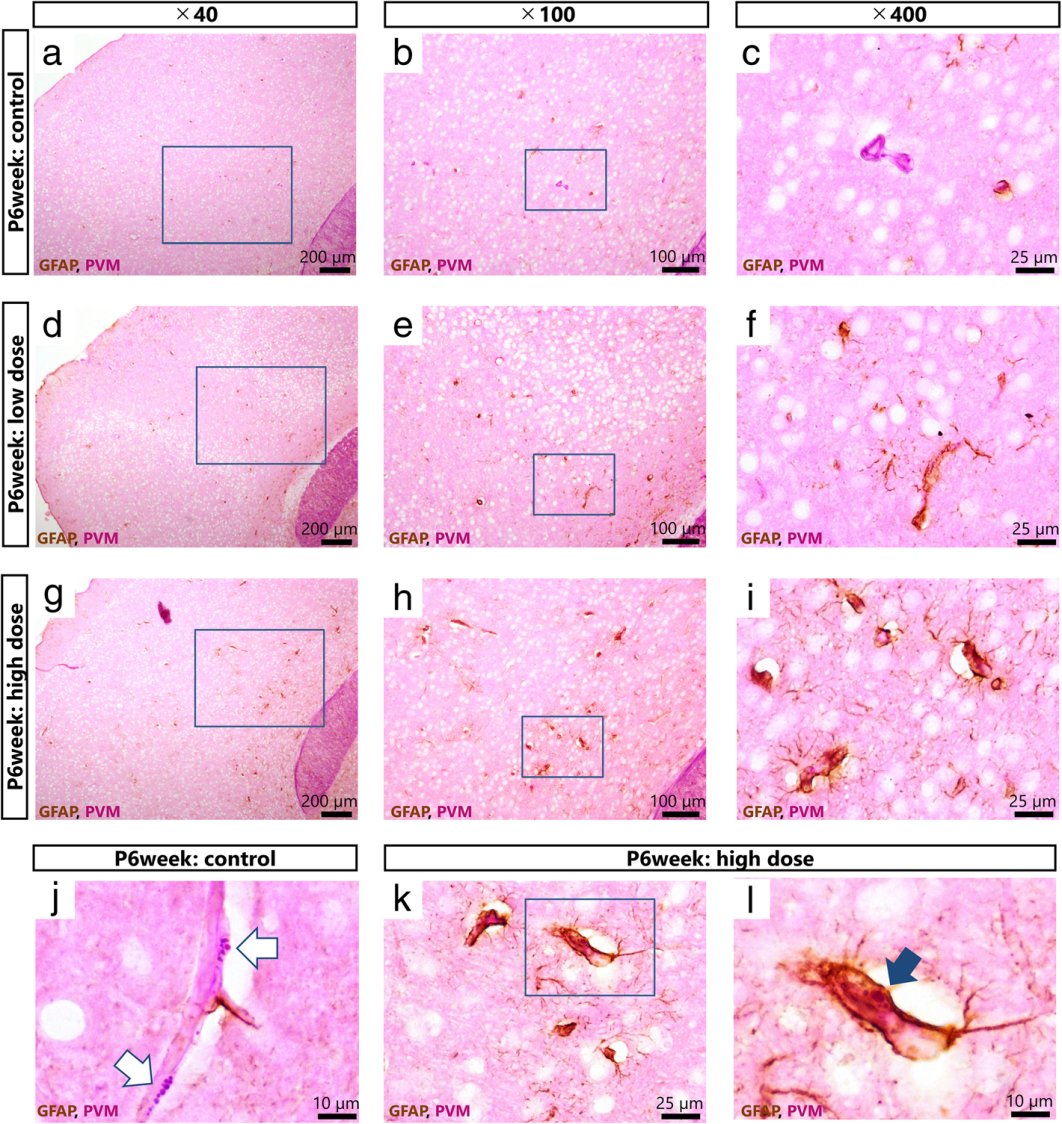
Maternal exposure to Printex 90 carbon nanoparticles affects astrocytes and perivascular macrophages (PVMs) in the offspring cerebral cortex (*n* = 4–6). **a**-**b** show representative light micrographs of glial fibrillary acidic protein (GFAP)-positive astrocytes and PVMs in the cerebral cortices of 6 week old male offspring in the control (**a**-**c**, **j**), low exposure (**d**-**f**), and high exposure groups (**g**-**i**, **k**, **l**). **a**, **d** and **g** gives an overview of the cerebral cortex, with **b**, **e**, and **h** providing enlarged views hereof, respectively, which is further enlarged in **c**, **f**, and **i**, respectively. These enlarged panels were taken from the same location in all brains. **c**, **f**, and **i** show that the expression levels of GFAP around blood vessels are dose-dependently increased by maternal exposure to carbon black nanoparticles. **j** and **k** show representative light micrographs of granules in PVMs of 6 week old male offspring in the control and high exposure groups, respectively. **l** is an enlarged view of panel **k**. White arrows indicate normal granules and black arrow indicates enlarged granules of PVMs. **l** shows that the granules in PVMs next to GFAP positive astrocytes are enlarged. Enlarged granules represent a typical histopathological finding following denaturation of PVMs

**Fig. 4:**
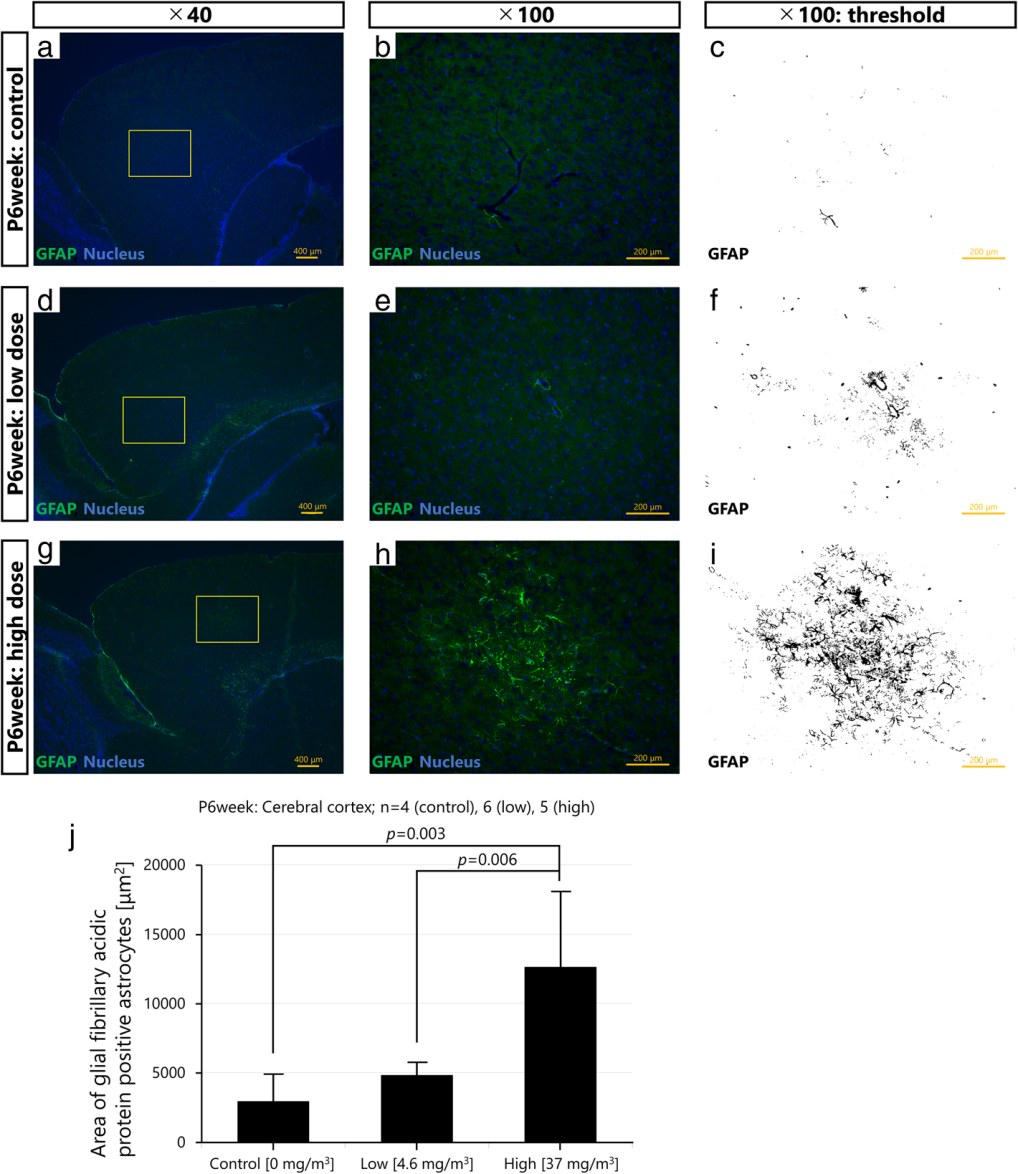
Maternal exposure to Printex 90 carbon nanoparticles dose-dependently increases expression levels of glial fibrillary acidic protein (GFAP) in astrocytes in the offspring cerebral cortex. **a**, **b**, **d**, **e**, **g** and **h** show representative fluorescent micrographs of GFAP-positive astrocytes in the cerebral cortices of 6 week old male offspring in the control (**a**, **b**), low exposure (**d**, **e**), and high exposure groups (**g**, **h**). **a**, **d** and **g** gives an overview of the cerebral cortex, with **b**, **e**, and **h** providing enlarged views hereof, respectively. **c**, **f**, and **i** are grayscale views of **b**, **e**, and **h**, respectively, for quantification of the GFAP expression. **j** indicates averages of the expression area of GFAP-positive astrocytes in the cerebral cortices of offspring based on the grayscale views provided in [Additional files 3, 4 and 5], and shows that the maternal exposure to the nanoparticles significantly increases expression levels of GFAP in astrocytes in offspring cerebral cortices. N is the number of brains analysed, mean ± SEM

**Fig. 5:**
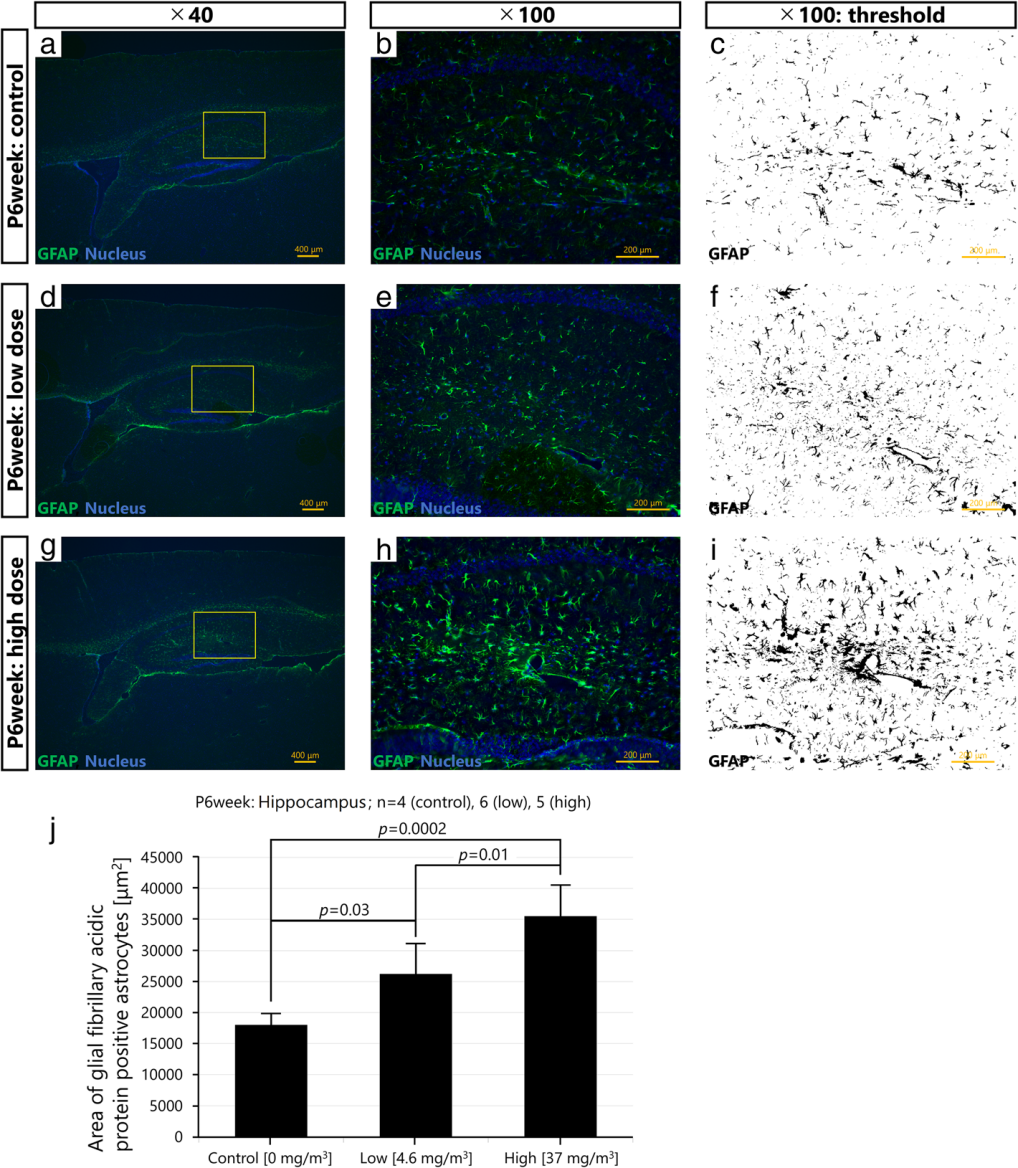
Maternal exposure to Printex 90 carbon nanoparticles dose-dependently increases expression levels of glial fibrillary acidic protein (GFAP) in astrocytes in the offspring hippocampus. **a**, **b**, **d**, **e**, **g** and **h** show representative fluorescent micrographs of GFAP-positive astrocytes in the hippocampi of 6 week old male offspring in the control (**a**, **b**), low exposure (**d**, **e**), and high exposure group (**g**, **h**). **a**, **d** and **g** gives an overview of the hippocampus, with **b**, **e**, and **h** providing enlarged views of panels **a**, **d**, and **g**, respectively. **c**, **f**, and **i** are grayscale views of **b**, **e**, and **h**, respectively, for quantification of GFAP expression. **j** indicates averages of the expression area of GFAP-positive astrocytes in the hippocampus of offspring based on grayscale views provided in [Additional files 6, 7 and 8], and shows that maternal exposure to the nanoparticles significantly increases expression levels of GFAP of astrocytes in the hippocampus. N is the number of brains analysed, mean ± SEM

First, the potential histopathological alterations of PVMs and astrocytes were assessed by PAS-GFAP immunohistochemistry. Inhalation exposure of pregnant mice to Printex 90 increased GFAP in the astrocytes around blood vessels in the offspring cerebral cortex. Enlarged lysosomal granules were also observed in the PVMs of these offspring (Fig. 3). Next, immunofluorescence analysis was performed to quantify GFAP protein levels in the offspring cerebral cortex and hippocampus. GFAP expression was statistically significantly increased in the cerebral cortex of the high exposure group compared to the low (*p* < 0.01) and the control (*p* < 0.01) exposure groups (Fig. 4 and Additional files 3, 4 and 5). GFAP levels were also significantly increased in the hippocampus of the high exposure group compared to the same region of the low (*p* < 0.05) and the control (*p* < 0.01) exposure groups, and in the low compared to the control exposure group (*p* < 0.05) (Fig. 5 and Additional files 6, 7 and 8). The increase in GFAP expression levels in the cerebral cortex and hippocampus increased in a dose-dependent manner following inhalation exposure to Printex 90 during pregnancy.

### Effects on parvalbumin positive interneurons in the cortex of the offspring

**Fig. 6:**
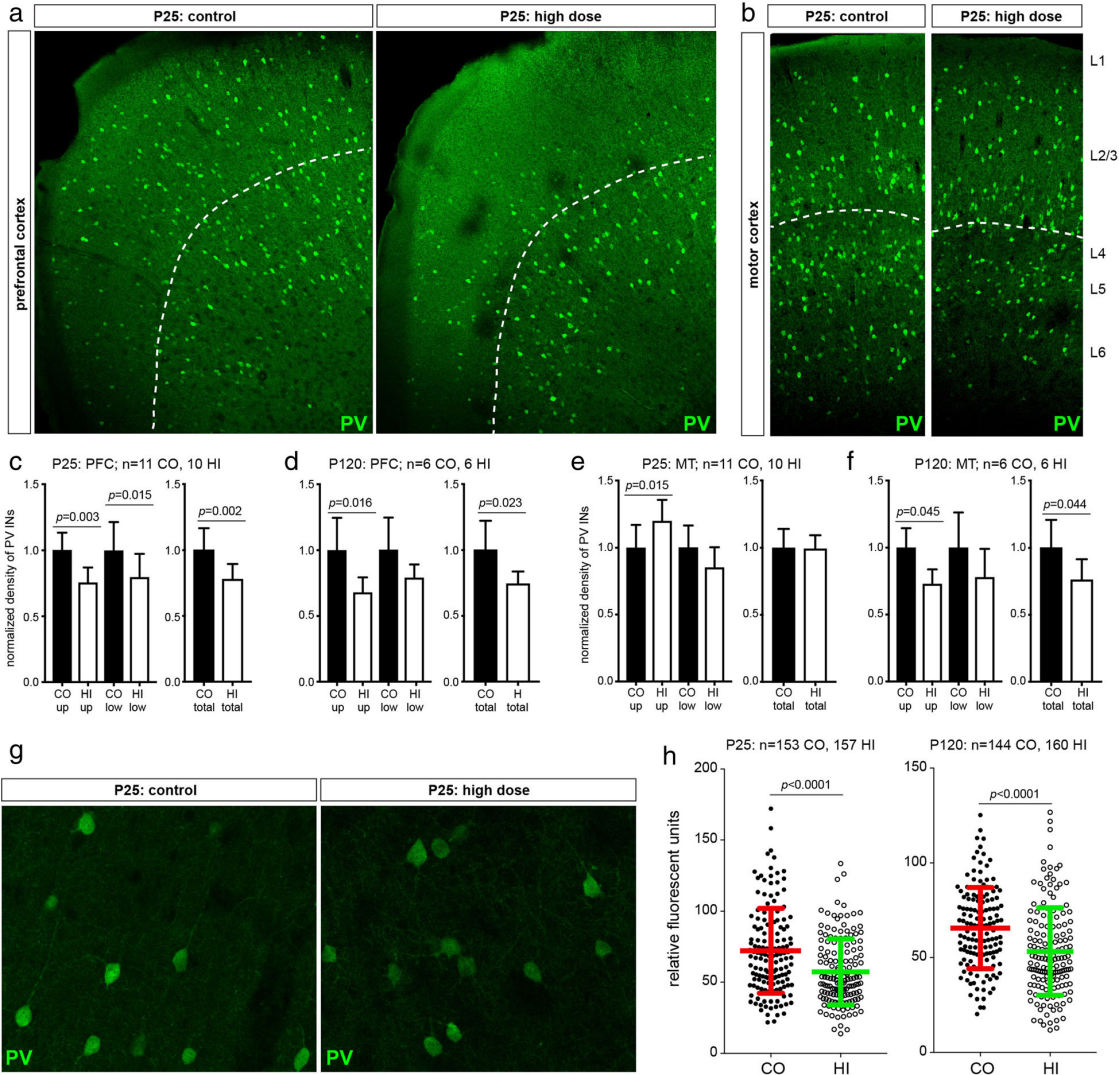
Maternal exposure to Printex 90 carbon nanoparticles affects parvalbumin (PV) expressing interneurons in the cortex. **a-f**: The density of PV interneurons is changed in the prefrontal (PFC) and motor (MT) cortices of the offspring following maternal exposure to carbon black (only control (CO) and high (HI) exposure groups investigated). **a** and **b** show an overview of the PFC and MT regions, respectively, which have been used for quantifications of PV interneurons. In both panels, the dashed line indicates separation of the upper (up; L1-L3) and lower (low; L4–6) cortical layers. **c** and **d** show that the density of PV interneurons in the PFC is decreased in both PND 25 and PND 120 offspring, respectively. The upper and lower layers of the PFC are similarly affected. **e** and **f**: The density of PV interneurons in the MT is affected by the maternal exposure. At PND 25 (**e**), there is an increase in the density of PV interneurons in the upper layers and a trend towards decrease (*p* = 0.078) in the lower layers at the highest maternal exposure level. At PND 120 (**f**) both upper and lower layers exhibit similar decreases in the density of PV interneurons. In all quantifications, the density of PV interneurons in control mice was normalized to 1 and the exposure group was normalized to the corresponding controls. N is the number of brains analysed. Mean ± SD. **g**-**h**: Expression of PV is decreased in the MT of maternally particle exposed offspring. **g** shows representative images of PV interneurons that were acquired without saturating the fluorescence signal. **h** shows that in prenatally exposed offspring PV interneurons have a dramatically decreased expression of PV both at PND 25 and PND 120. N denotes the number of cells measured

Maturation of PV+ interneurons was assessed in the prefrontal and motor cortices in animals from the control and the high exposure groups. At PND 25, both sexes were analysed and since there were no differences between the sexes, the data was combined. Already at PND 25, significantly fewer PV+ interneurons were detected in the prefrontal cortex in the high exposure group compared to control offspring, both in the upper and in lower cortical layers (Fig. 6a, c). This decrease in PV+ interneurons could also be observed at PND 120 (Fig. 6d). Interestingly, in the motor cortex, the upper and lower layers were differentially affected at PND 25 – more PV+ interneurons were detected in the upper layers of the prenatally highly exposed offspring, whereas there was a trend (*p* = 0.078) towards fewer PV+ interneurons in the lower layer, relative to control offspring (Fig. 6b, e). These findings emphasize the widespread and region-specific effects of Printex 90 in the offspring brain. By PND 120, the number of PV+ interneurons in the motor cortex was decreased in both the upper and lower layers in the prenatally exposed high-dose compared to control offspring (Fig. 6f). Furthermore, the expression of PV+ itself was heavily reduced in the prefrontal and motor cortices (Fig. 6g, h).

### Behaviour in the open field test

**Fig. 7:**
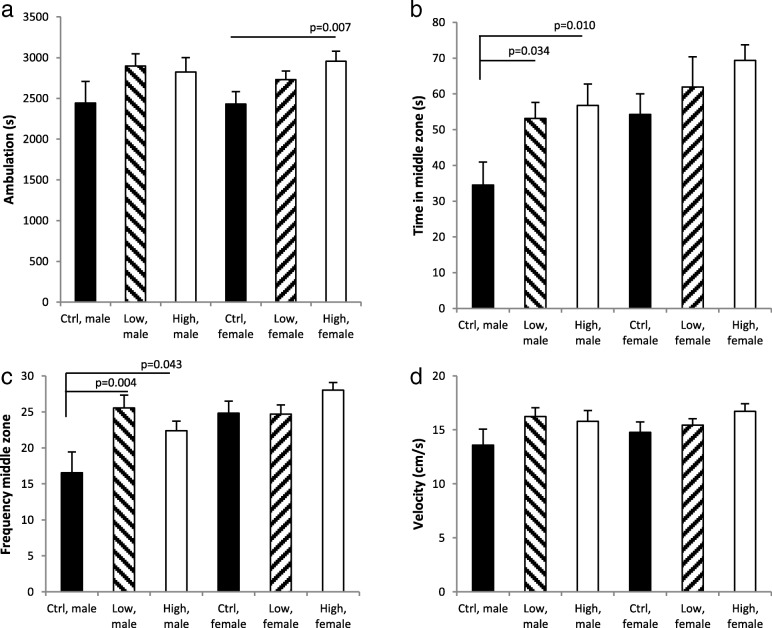
Maternal exposure to carbon black nanoparticles affects behaviour in the open field test during a 3 min observation period in male and female offspring. **a** shows the total ambulation during the 3 min test period where, overall, exposed offspring moved a longer distance than control offspring. **b** shows time spent in the central zone of field, where prenatally exposed offspring overall spent significantly longer time than exposed offspring. **c** shows the number of visits to the central zone, showing that prenatally exposed offspring visited the central zone more frequently than frequently than control offspring. **d** gives the velocity of ambulation. Mean ± SEM, *n* = 9–13

In the open field test, overall prenatally exposed animals moved a longer total distance than control offspring (*p* < 0.05) (Fig. 7a). Separating analysis by sex did however show that maternal exposure was significantly associated with distance moved only in females (*p* = 0.025), and differed statistically significantly between offspring from control and highly exposed mothers (*p* = 0.007). Overall, prenatally exposed animals also spent significantly longer time in the central zone of the field (*p* = 0.008, Fig. 7b), a parameter that furthermore differed significantly by sex (*p* = 0.006) probably reflecting the generally longer period of time spent centrally in the field by females, irrespective of exposure group. Albeit the pattern of effect was almost indistinguishable between males and females, time in the central zone differed significantly only in males (*p* = 0.026). Both the low and the high prenatal exposure males spent significantly more time in the central zone than control males (*p* = 0.034 and 0.010, respectively). Prenatally exposed offspring furthermore visited the central zone significantly more frequently (Exposure *p* = 0.005, Sex: *p* = 0.001; Exposure*Sex: *p* = 0.028). Separate analysis by sex indicated a significant effect of maternal exposure for males (*p* = 0.009; females: *p* = 0.096). Male offspring in both the low and the high exposure groups entered the central zone more frequently than control males (Fig. 7c, *p* = 0.0044 and 0.043, respectively).

## Discussion

The present study investigated offspring brain development following maternal inhalation of 4.8 or 33.9 mg Printex 90/m^3^, 45 min/day, on GD 4–18. Maternal exposure was associated with alterations in the histology of several cell populations in the CNS as well as in open field behaviour in the offspring. These changes were observed using a relevant route of maternal exposure (inhalation) and relevant exposure levels, as they corresponded to approximately 1 or 8 h, respectively, at the 8 h time weighted occupational exposure limit according to Danish Regulations [46]. The lowest observed adverse exposure concentration in the present study was therefore well below the allowed exposure level in the occupational setting.

Exposure was not associated with maternal pulmonary inflammation at either exposure level, when assessed 11 and 28–29 days after last exposure. Carbon black induced pulmonary inflammation decreases in a time dependent manner after termination of exposure [56, 57]. In a time series study, neutrophil influx was 76% decreased at 14 days post-exposure compared to one day post-exposure, where neutrophil influx was highest [56]. It is possible that the current exposure levels induced inflammation during and immediately after exposure but that inflammation had ceased at the time of measurement. In previous sub-chronic inhalation studies, pulmonary inflammation has been observed in rats exposed to 7.1 and 52.8 mg CB/m^3^ (6 h/day, 5 days/week for 13 weeks), but not to 1.1 mg/m^3^ [58]. Hence, while a daily dose of 6 h*1.1 mg/m^3^ (6.6 mg/m^3^) did not induce inflammation, daily exposure to 6*7.1 mg/m^3^ (43 mg/m^3^) did. This agrees well with our previous findings of significantly increased neutrophil influx in BALF 9 and 28–29 days after termination of inhalation exposure of pregnant mice to 42 mg/m^3^ Printex 90, 1 h/day for 11 days [42], but not to inhalation of 20 mg/m^3^ Printex 90 for 1.5 h/day for 4 consecutive days, measured immediately after end of exposure [59]. The daily exposure in the latter study – 1.5 h*20 mg/m^3^ (30 mg/m^3^) corresponds to the highest exposure level in the current study: 0.75 h*33.9 mg/m^3^ (25 mg/m^3^).

We assessed inflammation in two ways, by neutrophil influx into bronchoalveolar lavage fluid and by *Saa3* mRNA levels in lung tissue. In a study of the global transcription pattern, *Saa3* was the most differentially regulated gene in lung tissue both five days after inhalation of TiO_2_ NPs [60] and following pulmonary exposure to carbon black Printex 90 [56]. Furthermore, *Saa3* mRNA levels have consistently been shown to correlate closely with neutrophil influx across particle types, time points, doses and pulmonary exposure methods [61, 62].

Maternal inhalation of Printex 90 enlarged the lysosomal granules of PVMs in the brain of 6 weeks old male offspring. Similar findings have been observed following intranasal instillation of pregnant mice to Printex 90 at GD 5 and 9, where mild denaturation of PVMs and GFAP up-regulation in surrounding astrocytes were observed in 6 and 12 week old males at maternal dose levels of 15, 73, and 95 μg/kg/day, but not 2.9 μg/kg/day [36, 37]. The current study quantitatively evaluated GFAP expression by immunofluorescence, and its increase was confirmed in the cerebral cortex and hippocampus, both regions studied. It is possible that these changes were more widespread, as the perivascular changes characterized by long-term PVM denaturation have previously been observed diffusely in grey matter throughout the brain [36]. PVMs adjoin the endothelial cells, the pericytes, and the endfeet of astrocytes, extending to neighbouring blood vessels to participate in the formation and maintenance of the blood-brain barrier [63]. During injury, astrogliosis may facilitate the reconstruction of the blood-brain barrier and tissue remodelling [64, 65]. Our observation of increased GFAP expression suggests that inhalation exposure to Printex 90 during pregnancy may induce chronic astrocyte activation. Upregulation of GFAP generally occurs when the nervous system is damaged [66], but how astrocytes are activated is so far unknown [17]. Many previous studies have shown that such reactive astrogliosis is a general feature of brain ageing [67–69]. Hence, PVM granules generally enlarge with increasing age, presumably due to uptake and accumulation of substances from plasma [70]. Accordingly, the observed changes may constitute a sign of premature brain ageing, and, hypothetically, predispose the offspring to age-related brain disorders. Interestingly, findings in a recent cohort study suggest that long-term exposure to ambient fine particles in adulthood may accelerate the effects of aging in the brain [71].

Astrogliosis has also been observed following other types of maternal conditions during gestation, such as maternal immune activation by LPS and maternal obesity [72–74]. In mice, maternal obesity induced by consumption of high-fat diet was shown to stimulate proliferation of GFAP positive astrocytes in the hypothalamus, possibly mediated by increased interleukin-6 (IL-6) in the foetal circulation [74, 75]. In addition to upregulation of GFAP, also upregulation of CD206 in PVMs, a marker of abnormal drainage of amyloid-β in the brain, has been observed in the adult offspring of dams fed a high-fat diet [73].

Several studies suggest that PV+ interneurons in the cortex and the hippocampus are especially vulnerable to maternal inflammation or oxidative stress [26–29]. In the present study, the number of PV+ interneurons was reduced in the cortex and the expression of PV was decreased in the offspring of mothers exposed to Printex 90. Since PV acts as a calcium buffer, with an important role in synaptic plasticity [76], the functioning of PV+ interneurons in offspring from the highest exposed dams are likely to be compromised, as was shown for PV+ interneurons in the offspring affected by maternal inflammation induced by poly(I:C) [28]. The heavily reduced expression of PV+ itself in the prefrontal and motor cortices are furthermore indicative of a schizophrenia-like phenotype [77–80].

Overall, the changes in PV interneurons bear high resemblance to the observations in established animal models of maternal inflammation [26, 27, 29]. As previously discussed, we were unable to detect maternal inflammation 11 and 28–29 days after exposure, but we cannot rule out that inflammation was present at earlier time points. Maternal Printex 90 exposure activated astrocytes in the offspring brain. Previous studies have shown that the activated astrocyte is a main source of the pro-inflammatory cytokine IL-6 in the central nervous system [81]. Importantly, IL-6 is the major pro-inflammatory molecule in several mouse models of maternal inflammation. In these models, IL-6 increases abruptly immediately upon maternal immune activation and contributes to the pathological development of the foetal brain [25]. We have previously found increased IL-6 mRNA levels in lung tissue 3 h after pulmonary exposure to Printex 90 but not at later time points [56], and increased IL-6 protein levels in lung tissue in dams with litters 26–27 days following intratracheal instillation of a total of 268 μg of Printex 90 but not at lower dose levels [32]. Secretion of IL-6 from activated astrocytes in the foetal brain in the current study might have acted similarly to the increase in IL-6 levels in models of maternal inflammation. PV+ interneurons might be a common target for inflammatory mediators in developing offspring, whether mediators originate from maternal inflammation or locally in the brain. Future studies ought to include measurement of IL-6 in offspring brains.

In the open field test, maternal exposure to Printex 90 changed offspring behaviour. Visual inspection of Fig. 7 indicated a dose-dependent (albeit not always) pattern of effect that to some degree repeated itself between females and males. The latter was confirmed by the presence of interaction between exposure and sex only for the frequency of visits to the central zone of the field. The statistical analysis did however show that gestationally exposed females mainly moved a longer distance, whereas males rummaged significantly more in the central maze zone. Sex differences in behaviour are often observed following changes in the intrauterine environment [82], and this is also the case following maternal exposure to particles [3]. At present the toxicological database does not suffice to evaluate whether this is a general issue for maternal particulate exposures. It is however also possible that the time courses in development of relevant neural structures differed between males and females relative to the time points of maternal exposure and that this could contribute to the slight dfferences between the sexes [83, 84]. We also previously found female offspring from pregnant mice inhaling 19 mg/m^3^ diesel exhaust particles (NIST SRM 2975), 1 h/day at GD 9–19, to display a tendency towards increased activity in the open field (time in the central zone not assessed; [44]). Gestational instillation of a total of 268 μg Printex 90 altered the habituation pattern in female offspring [41]. In contrast, instillation of female mice with 67 μg of MWCNT (NM-400) on the day prior to mating did not change open field activity in 3 months old male offspring (females not tested), even though the exposed females most likely experienced inflammation during the gestational period [85]. Gestational inhalation of TiO_2_ UV-Titan L181 NP (42 mg/m^3^, 1 h/day at GD 9–18) was associated with a significant avoidance of the central zone in both sexes [45]. In most of the previous studies, airway exposure caused significant maternal lung inflammation [41, 45, 85], but overall, maternal lung inflammation does not seem associated with nor required for changes in open field behaviour. The different findings for prenatal exposure to UV-Titan L181 NP as compared to Printex 90 indicate that the particles (or associated chemical constituents) may have interfered with brain development in a direct way [3, 18]. As the present exposure was via inhalation, some possible neurotoxic impact of the inhalation to the dams (via the olfactory pathway, with possible translocation of CB-NP to the brain of the dams) cannot be excluded. Hence impact on offspring brain development could potentially originate from the sequelae of particle induced neurological impact in the maternal mice [17, 30]. Offspring from the maternal inflammation model of injected poly(I:C) has been shown to generally spend less rather than more time in the central zone of the open field [86]. In spite of the similarities in effect on inhibitory interneurons between this and exposure to Printex 90, effects on behaviour might differ between the various models. PV+ interneurons exert rigorous control over activity in the prefrontal cortex, but little is known about their control over behavioral functions. In adult mice, blocking of the output from PV+ interneurons left locomotion and anxiety related behavior unaltered, and rather interfered with execution of working memory functions [87]. Whether the reduced density of PV+ interneurons and expression of PV are interrelated with the behavioural changes in the present study remains speculative for now. It would be interesting to compare the effects on inhibitory interneurons, PVMs, astrocytes and relevant behavioural domains, such as the basal acoustic startle reaction and pre-pulse inhibition, cognitive function and social behaviour, across maternal exposure to CB-NP, poly(I:C) and obesity induced by high fat diet [3, 29, 88].

Timing of exposure also constitutes a major difference between the described studies. Exposure started at GD 4 in the present compared to GD 7–9 in the other studies, including those of poly(I:C). Initiation of exposure as early as at GD 4 may therefore have interfered with the extensive epigenetic reorganization initiated prior to implantation [89, 90] and the establishment of astrocytic and PVM populations in the developing brain. PVMs translocate from progenitor cells in the yolk sac at GD 7.5, and persist in the brain throughout life [91, 92]. If the behavioural changes in the present study arose due to disturbance of the very early developmental steps, then initiation of exposure at GD 7–9 may have been too late for the present pattern to manifest. Furthermore, if similar mechanisms operate in humans, it implies that exposure to carbonaceous particles should be minimized very early in pregnancy, and maybe even prior to fertilization. The potential implication of periconceptional exposures is an important point to pursue in future work.

The induction of denaturation of the PVMs and of reactive astrocytes by maternal Printex 90 exposure seems very robust, as dose-dependent effects now have been observed following maternal exposure via inhalation as well as via intranasal instillation, and in outbred as well as inbred strains of mice. Importantly, the present findings confirmed that proof-of-principle studies using intratracheal and intranasal instillation can be used to identify effects that arise after inhalation, the golden standard of exposure.

The present study has several limitations. Markers of inflammation were measured only sometime after termination of exposure and only in the exposed adult females. The level of inflammation, if any, during foetal development or in the offspring after birth is therefore not known. The lack of a lower dose level with no observed effects in the offspring precludes definition of a limit values based on the no observed adverse effect level. The different outcomes were assessed at different time-points, precluding correlation analysis hereof, and the study would have benefited from a more extensive battery of behavioural tests.

## Conclusions

The present study demonstrates that maternal inhalation exposure to Printex 90 interferes dose-dependently with brain development and that maternal inhalation exposure to Printex 90 in the offspring brain induces similar effects as previously observed following carbon black exposure by intratracheal and intranasal instillation. Of note, the observed effects have striking similarities with those observed in mouse models of neurodevelopmental disorders. The observation of effects at exposure levels below the existing occupational exposure limit calls for re-evaluation hereof.

## Additional files

Additional file 1: Experimental setup for the animal exposure. Time-mated animals were placed in separate rooms of a cylindrical wire mesh cage (∅ 29 cm, height 9 cm) with twelve rooms arranged in radial partitions, placed in an 18 L spheroidal chamber with a stainless steel rim and upper and lower spheres in fully transparent glass, designed to provide an evenly distributed exposure atmosphere. Printex 90 was aerosolized using a rotating disc microfeeder and directed through an aerosol mixing and sedimentation glass-tube to the exposure chamber. Mass-concentrations of total suspended dust were controlled by filter sampling. Particle number and aerodynamic particle size distributions in the particle exposure atmosphere were measured using an Electrical Low Pressure Impactor. Sham exposure for control animals was monitored using a condensation particle counter. (PNG 59 kb)

Additional file 2: Total particle number concentration during the exposure period. Insert shows an example of a typical exposure during a single day, measured by the ELPI+ that was running all day. Full and dashed lines signifies start and end of exposure, respectively, with two groups of low exposure in the morning and two groups of high exposure in the afternoon. Control groups were run alongside exposed groups in a separate chamber. (PNG 148 kb)

Additional file 3: Fluorescent micrographs and converted grayscale views of GFAP-positive astrocytes in the cerebral cortices of control offspring (*n* = 4). Three pictures are randomly (with > 30 μm interval) chosen from the brain of each male offspring. Twelve selected images are converted to grayscale views for quantification of the GFAP expression. (TIF 5914 kb)

Additional file 4: Fluorescent micrographs and converted grayscale views of GFAP-positive astrocytes in the cerebral cortices of offspring from low-dose dams (*n* = 6). Three pictures are randomly (with > 30 μm interval) chosen from the brain of each male offspring. Eighteen selected images are converted to grayscale views for quantification of the GFAP expression. (TIF 8857 kb)

Additional file 5: Fluorescent micrographs and converted grayscale views of GFAP-positive astrocytes in the cerebral cortices of offspring from high-dose dams (*n* = 5). Three pictures are randomly (with > 30 μm interval) chosen from the brain of each male offspring. Fifteen selected images are converted to grayscale views for quantification of the GFAP expression. (TIF 8176 kb)

Additional file 6: Fluorescent micrographs and converted grayscale views of GFAP-positive astrocytes in the hippocampus of control offspring (*n* = 4). Three pictures are randomly (with > 30 μm interval) chosen from each brain of each offspring mouse. Twelve selected pictures are converted to grayscale views for quantification of the GFAP expression. (TIF 7341 kb)

Additional file 7: Fluorescent micrographs and converted grayscale views of GFAP-positive astrocytes in the hippocampus of offspring from low-dose dams (n = 6). Three pictures are randomly (with > 30 μm interval) chosen from each brain of each offspring mouse. Eighteen selected pictures are converted to grayscale views for quantification of the GFAP expression. (TIF 10835 kb)

Additional file 8: Fluorescent micrographs and converted grayscale views of GFAP-positive astrocytes in the hippocampus of offspring from high-dose dams (*n* = 5). Three pictures are randomly (with > 30 μm interval) chosen from each brain of each offspring mouse. Fifteen selected pictures are converted to grayscale views for quantification of the GFAP expression. (TIF 9712 kb)

## Abbreviations

∅: Diameter
ANOVA: Analysis of variance
BALF: Bronchoalveolar lavage fluid
BSA: Bovine serum albumin
CB-NP: Carbon black nanoparticles
CNS: Central nervous system
ELPI: Electrical low pressure impactor
GD: Gestation day
GFAP: Glial fibrillary acidic protein
IL-6: Interleukin 6
LPS: Lipopolysaccharide
MT: Motor cortex
NP: Nanoparticles
PAS: Periodic acid Schiff
PBS: Phosphate buffered saline
PFC: Prefrontal cortex
PND: Postnatal day
poly(I:C): Polyinosinic-polycytidilic acid
PV+: Parvalbumin-positive
PVM: Perivascular macrophage
SD: Standard deviation
SEM: Standard error of the mean
SMPS: Sequential (stepping) mobility particle sizer
TEM: Transmission electron microscopy
TIFF: Tagged image file format
TiO_2_: Titanium dioxide
