# Supplementary figures and images for: Maternal inhalation of carbon black nanoparticles induces neurodevelopmental changes in mouse offspring
Source: Part Fibre Toxicol. 2018 Sep 10;15:36. doi: 10.1186/s12989-018-0272-2 (PMC6131790; doi:10.1186/s12989-018-0272-2)

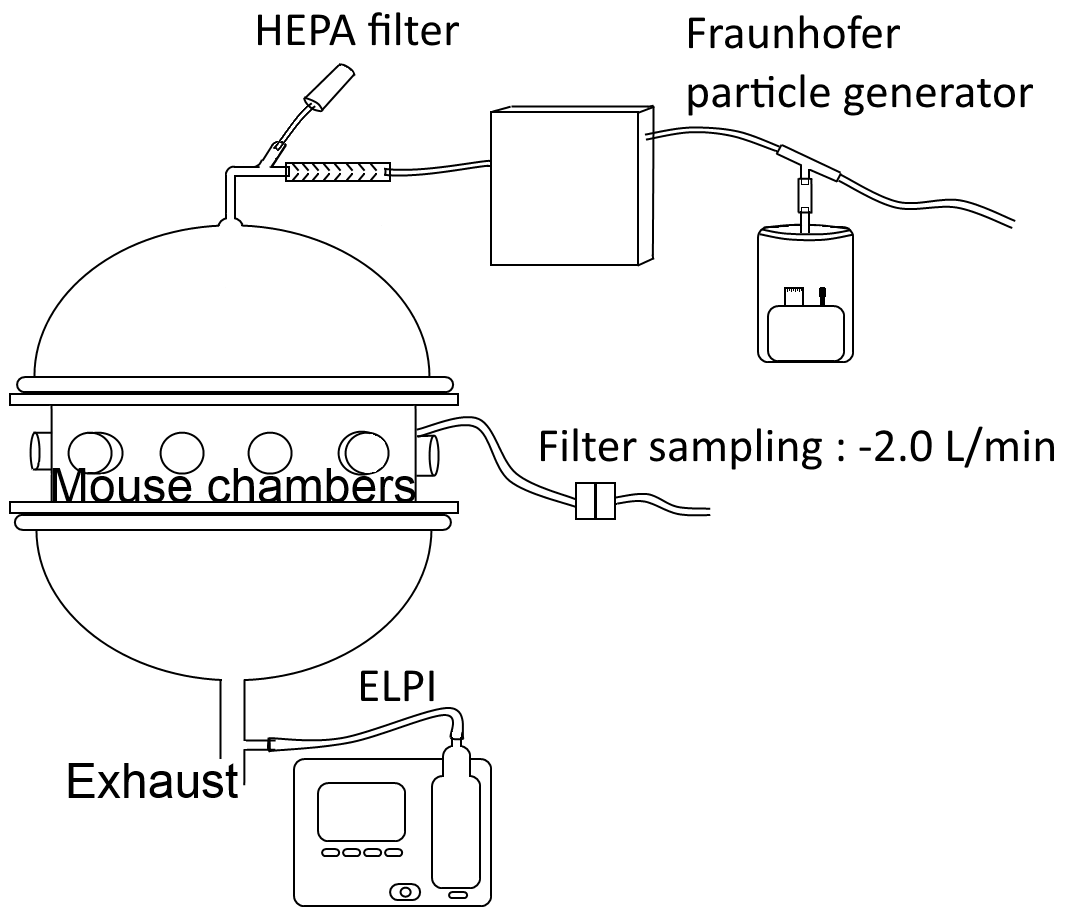

Supplement: Supplementary file 1 — Experimental setup for the animal exposure. Time-mated animals were placed in separate rooms of a cylindrical wire mesh cage (∅ 29 cm, height 9 cm) with twelve rooms arranged in radial partitions, placed in an 18 L spheroidal chamber with a stainless steel rim and upper and lower spheres in fully transparent glass, designed to provide an evenly distributed exposure atmosphere. Printex 90 was aerosolized using a rotating disc microfeeder and directed through an aerosol mixing and sedimentation glass-tube to the exposure chamber. Mass-concentrations of total suspended dust were controlled by filter sampling. Particle number and aerodynamic particle size distributions in the particle exposure atmosphere were measured using an Electrical Low Pressure Impactor. Sham exposure for control animals was monitored using a condensation particle counter. (PNG 59 kb) [file 12989_2018_272_MOESM1_ESM.png]

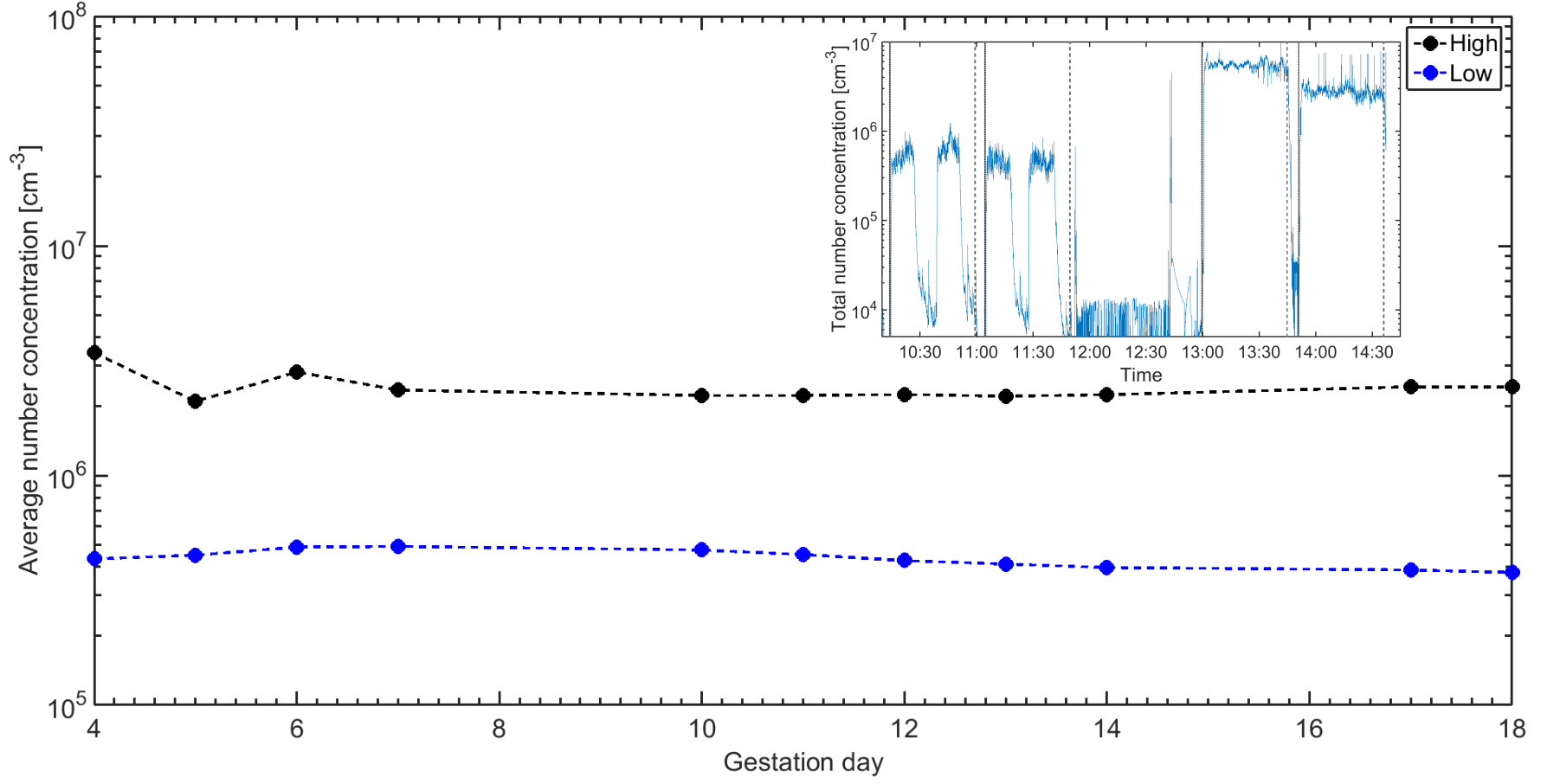

Supplement: Supplementary file 2 — Total particle number concentration during the exposure period. Insert shows an example of a typical exposure during a single day, measured by the ELPI+ that was running all day. Full and dashed lines signifies start and end of exposure, respectively, with two groups of low exposure in the morning and two groups of high exposure in the afternoon. Control groups were run alongside exposed groups in a separate chamber. (PNG 148 kb) [file 12989_2018_272_MOESM2_ESM.png]

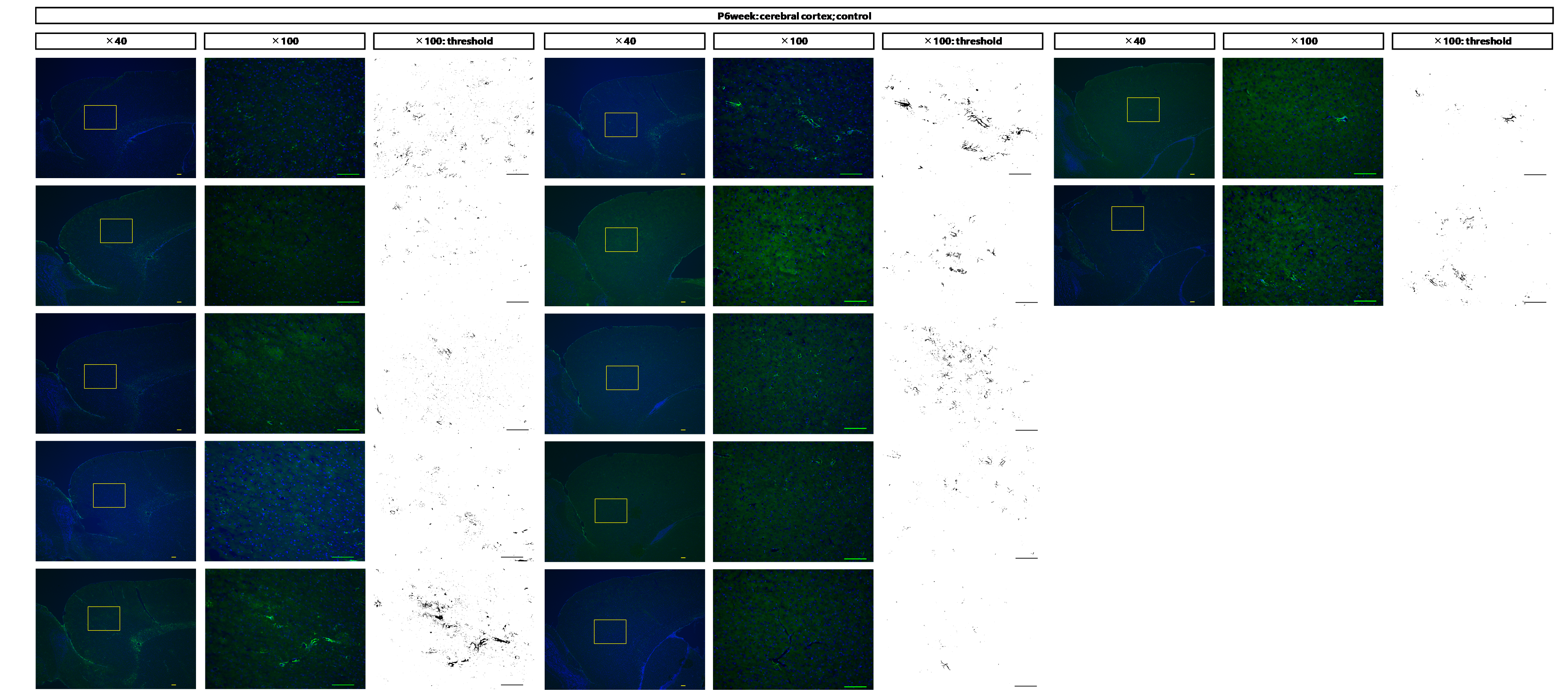

Supplement: Supplementary file 3 — Fluorescent micrographs and converted grayscale views of GFAP-positive astrocytes in the cerebral cortices of control offspring (n = 4). Three pictures are randomly (with > 30 μm interval) chosen from the brain of each male offspring. Twelve selected images are converted to grayscale views for quantification of the GFAP expression. (TIF 5914 kb) [file 12989_2018_272_MOESM3_ESM.tif]

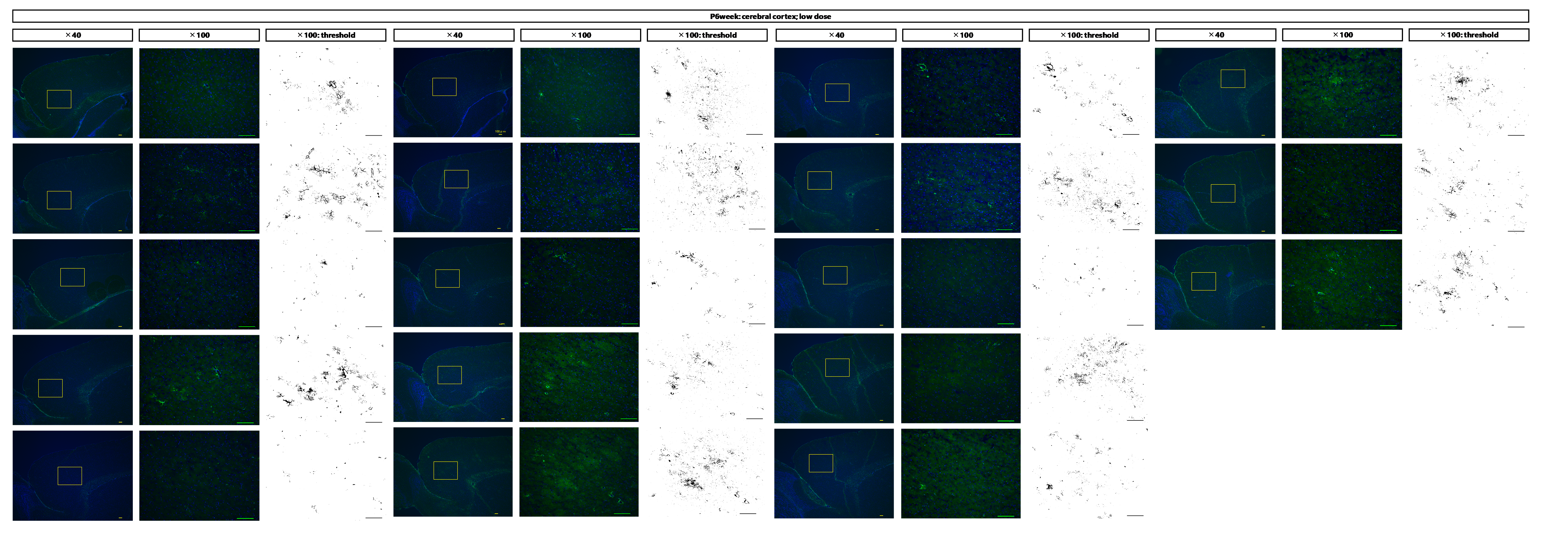

Supplement: Supplementary file 4 — Fluorescent micrographs and converted grayscale views of GFAP-positive astrocytes in the cerebral cortices of offspring from low-dose dams (n = 6). Three pictures are randomly (with > 30 μm interval) chosen from the brain of each male offspring. Eighteen selected images are converted to grayscale views for quantification of the GFAP expression. (TIF 8857 kb) [file 12989_2018_272_MOESM4_ESM.tif]

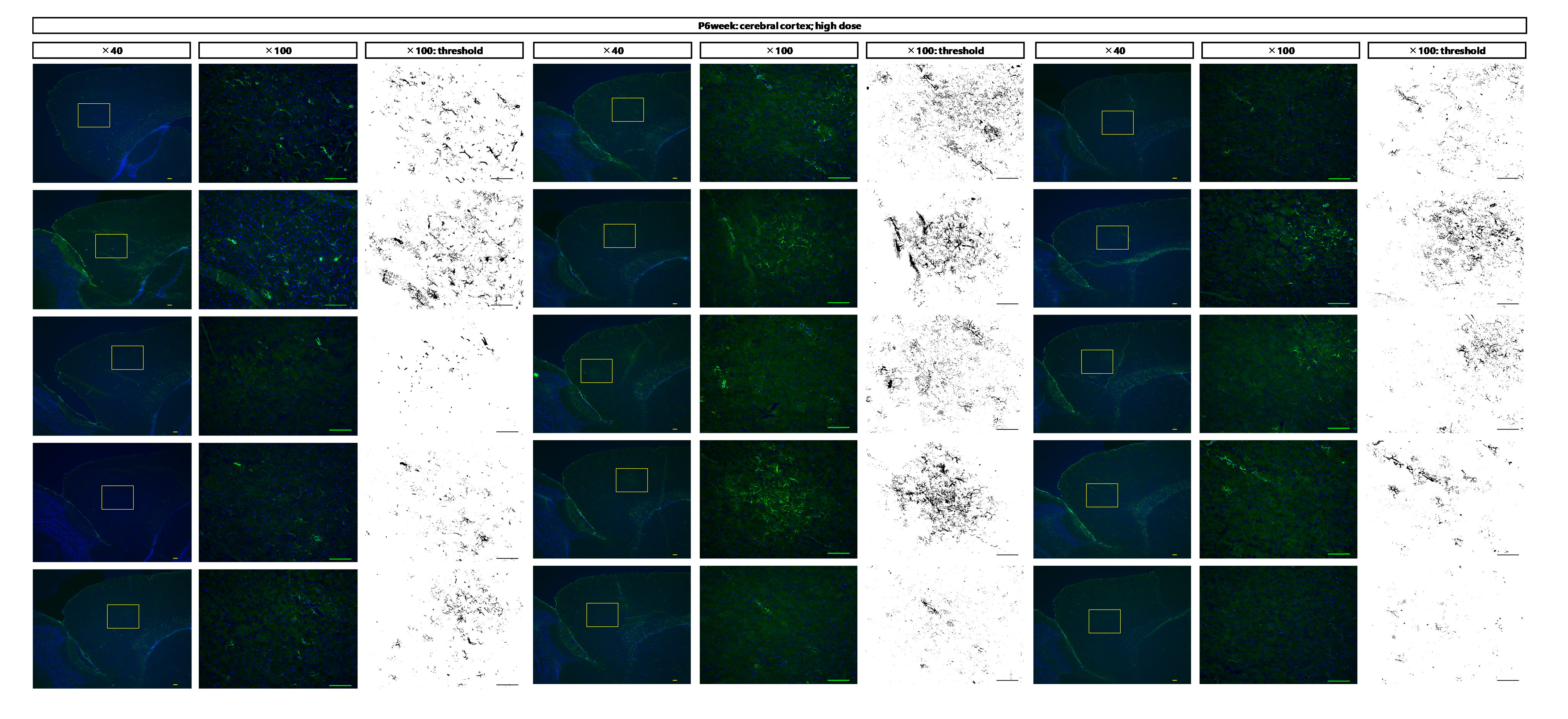

Supplement: Supplementary file 5 — Fluorescent micrographs and converted grayscale views of GFAP-positive astrocytes in the cerebral cortices of offspring from high-dose dams (n = 5). Three pictures are randomly (with > 30 μm interval) chosen from the brain of each male offspring. Fifteen selected images are converted to grayscale views for quantification of the GFAP expression. (TIF 8176 kb) [file 12989_2018_272_MOESM5_ESM.tif]

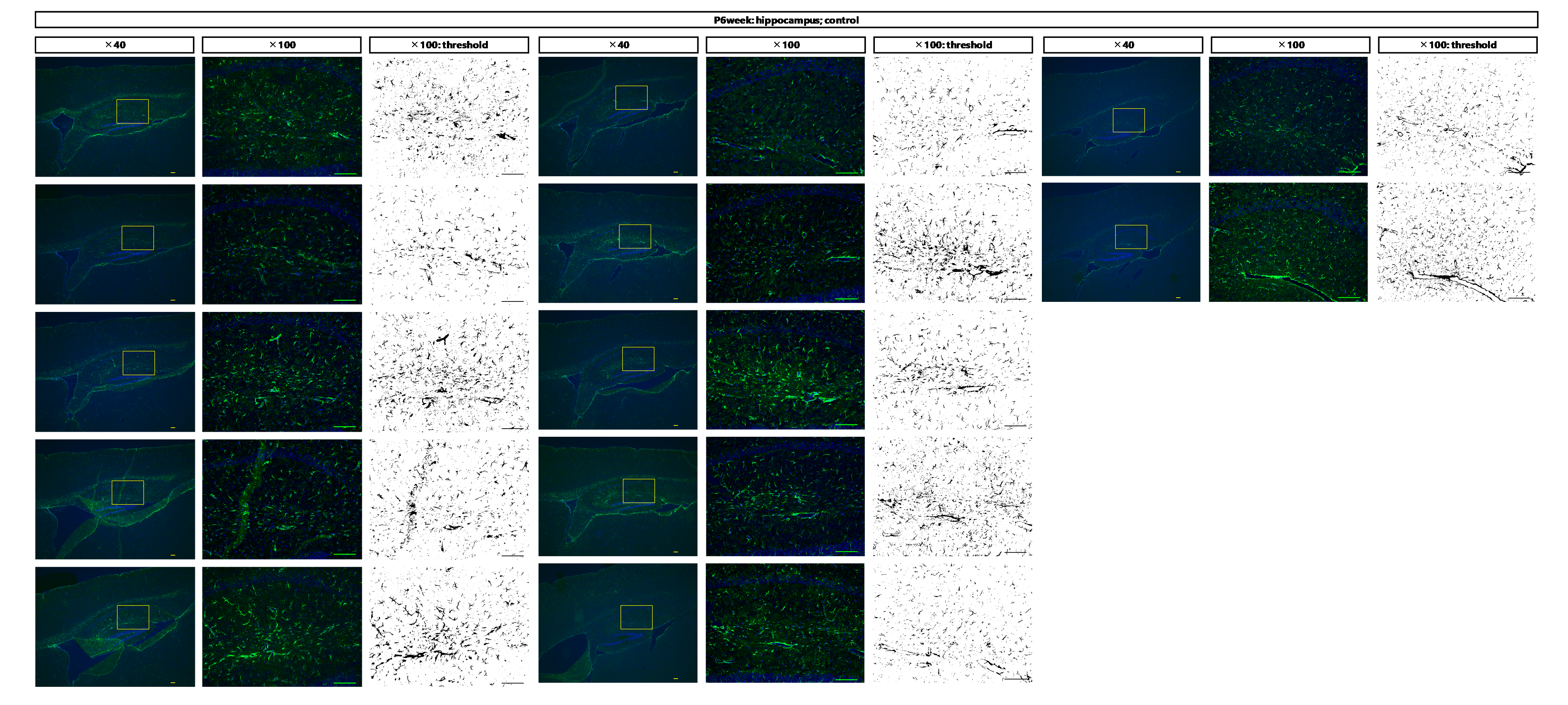

Supplement: Supplementary file 6 — Fluorescent micrographs and converted grayscale views of GFAP-positive astrocytes in the hippocampus of control offspring (n = 4). Three pictures are randomly (with > 30 μm interval) chosen from each brain of each offspring mouse. Twelve selected pictures are converted to grayscale views for quantification of the GFAP expression. (TIF 7341 kb) [file 12989_2018_272_MOESM6_ESM.tif]

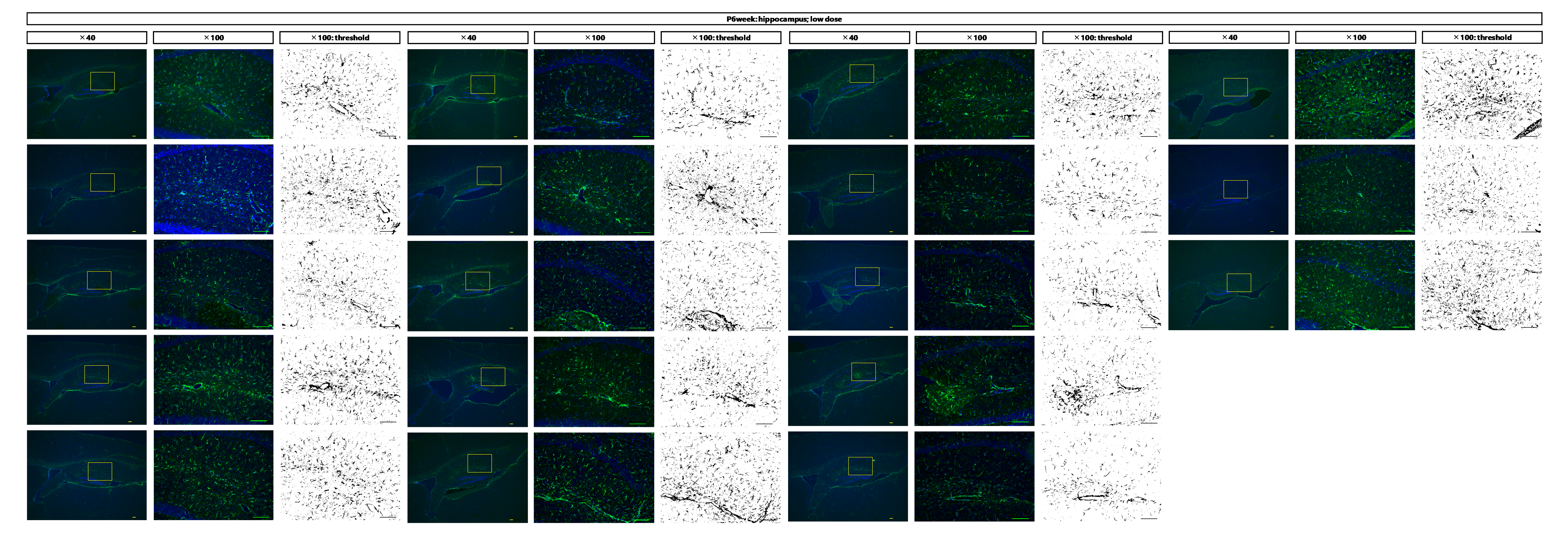

Supplement: Supplementary file 7 — Fluorescent micrographs and converted grayscale views of GFAP-positive astrocytes in the hippocampus of offspring from low-dose dams (n = 6). Three pictures are randomly (with > 30 μm interval) chosen from each brain of each offspring mouse. Eighteen selected pictures are converted to grayscale views for quantification of the GFAP expression. (TIF 10835 kb) [file 12989_2018_272_MOESM7_ESM.tif]

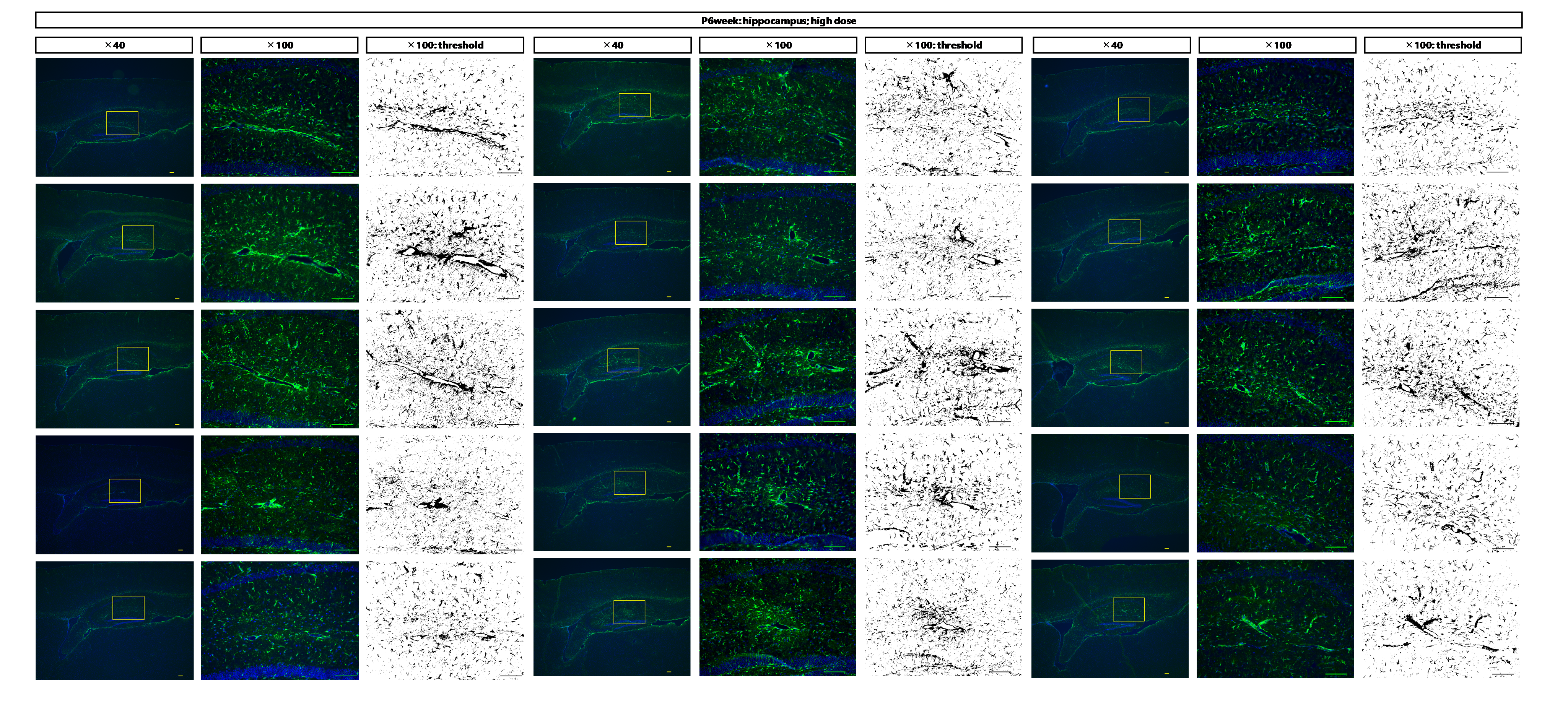

Supplement: Supplementary file 8 — Fluorescent micrographs and converted grayscale views of GFAP-positive astrocytes in the hippocampus of offspring from high-dose dams (n = 5). Three pictures are randomly (with > 30 μm interval) chosen from each brain of each offspring mouse. Fifteen selected pictures are converted to grayscale views for quantification of the GFAP expression. (TIF 9712 kb) [file 12989_2018_272_MOESM8_ESM.tif]
